# Supplementary material for: A nanoscale reciprocating rotary mechanism with coordinated mobility control
Source: Nat Commun. 2021 Dec 8;12:7138. doi: 10.1038/s41467-021-27230-7 (PMC8654862; doi:10.1038/s41467-021-27230-7)
Supplement: Supplementary file 1 — Supplementary Information [file 41467_2021_27230_MOESM1_ESM.pdf]

A nanoscale reciprocating rotary mechanism with coordinated mobility control

Eva Bertosin[1,2], Christopher M. Maffeo[3,4], Thomas Drexler[1,2], Maximilian N. Honemann[1,2], Aleksei Aksimentiev[3,4], Hendrik Dietz[1,2]

[1]Lehrstuhl für Biomolekulare Nanotechnologie, Physik Department, Technische Universität München, Garching near Munich, Germany

[2]Munich Institute of Biomedical Engineering, Technische Universität München, Garching near Munich, Germany

[3]Department of Physics, University of Illinois at Urbana-Champaign, Urbana, IL, 61801, USA

[4]Beckman Institute for Advanced Science and Technology, University of Illinois at Urbana-Champaign, Urbana, IL, 61801, USA

Correspondence to dietz@tum.de

Supplementary Information

Content

Supplementary Figures 1-34

Supplementary Table 1

Supplementary Figures

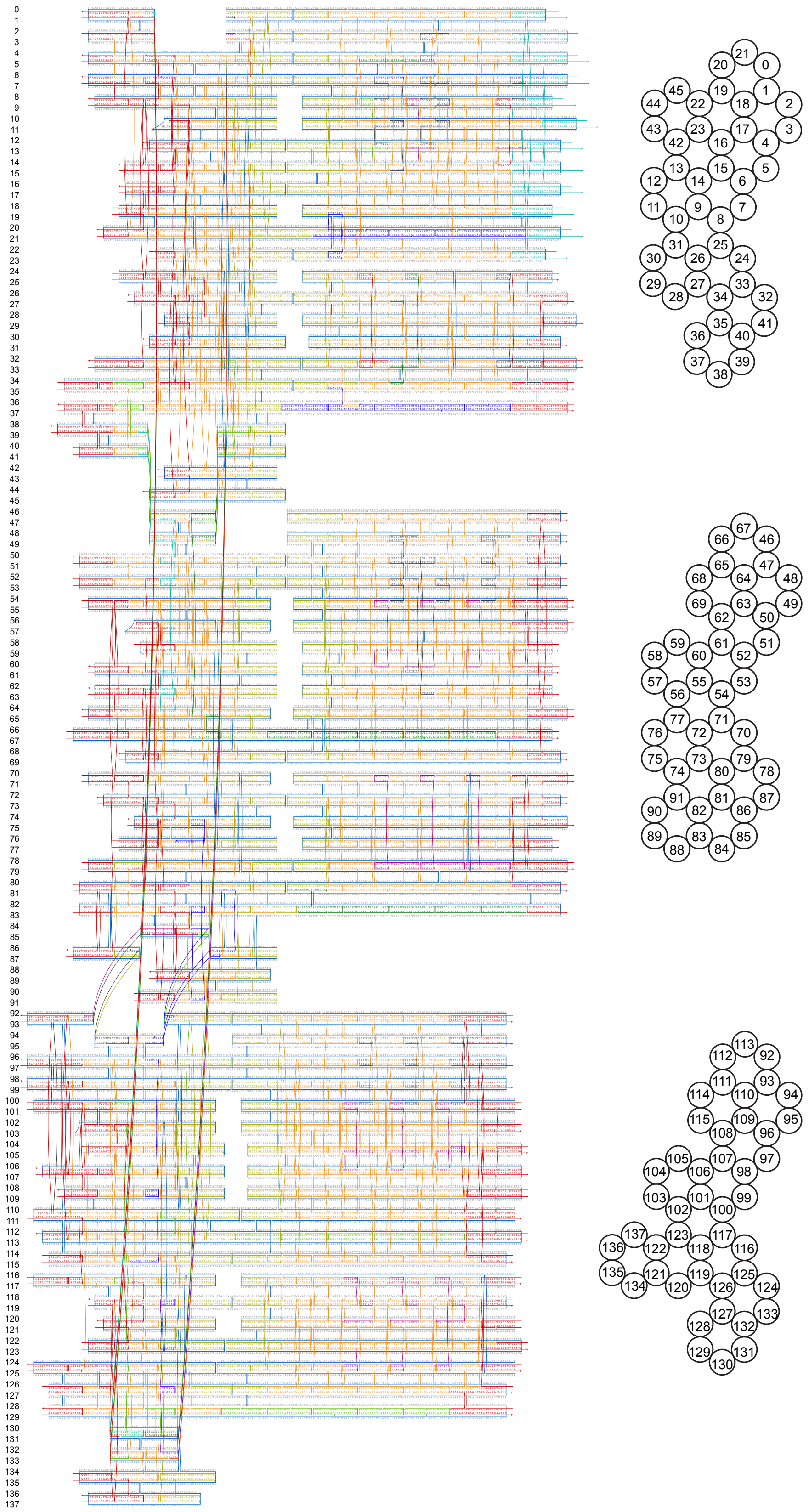

Supplementary Figure 1 | CaDNAno (*1*) design diagrams (left) and bottom view cross sections (right) of the stator units 1 (top), 2 (middle) and 3 (bottom).

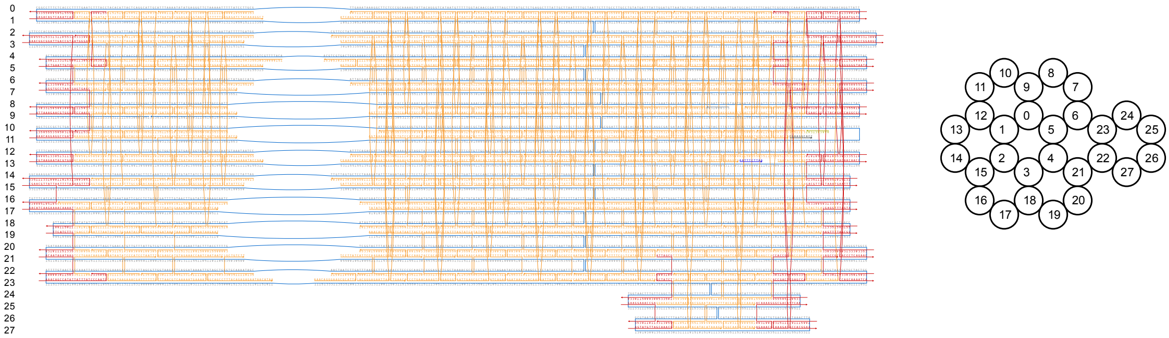

Supplementary Figure 2 | CaDNAno (*1*) design diagram (left) and bottom-view cross section (right) of the central camshaft.

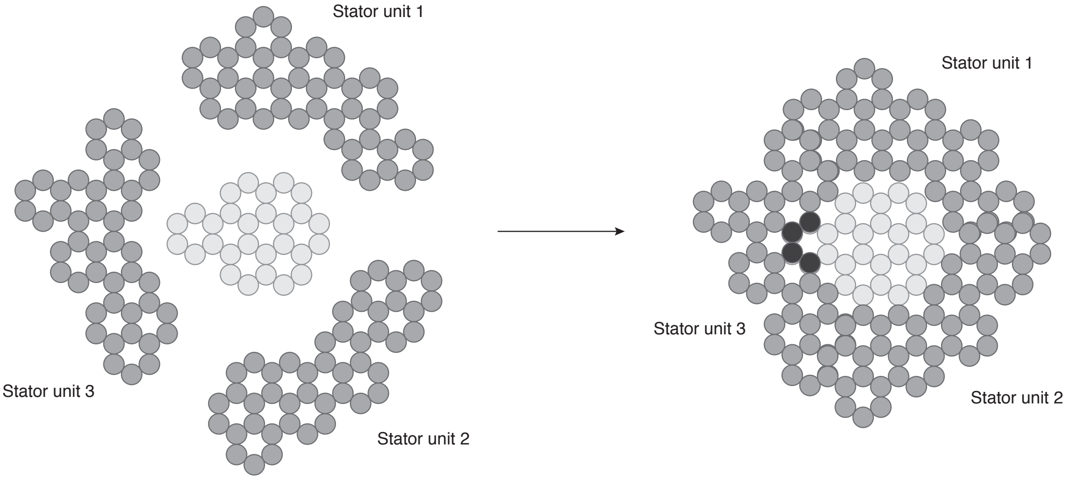

Supplementary Figure 3 | Schematic representation of the helical cross section of the rotary mechanism. Each circle represents a DNA double helix. Top view cross sections of the monomers composing the mechanism (left) and cross section of the fully formed mechanism (right). In black the helices of the camshaft that overlap with the helices of the surrounding stator.

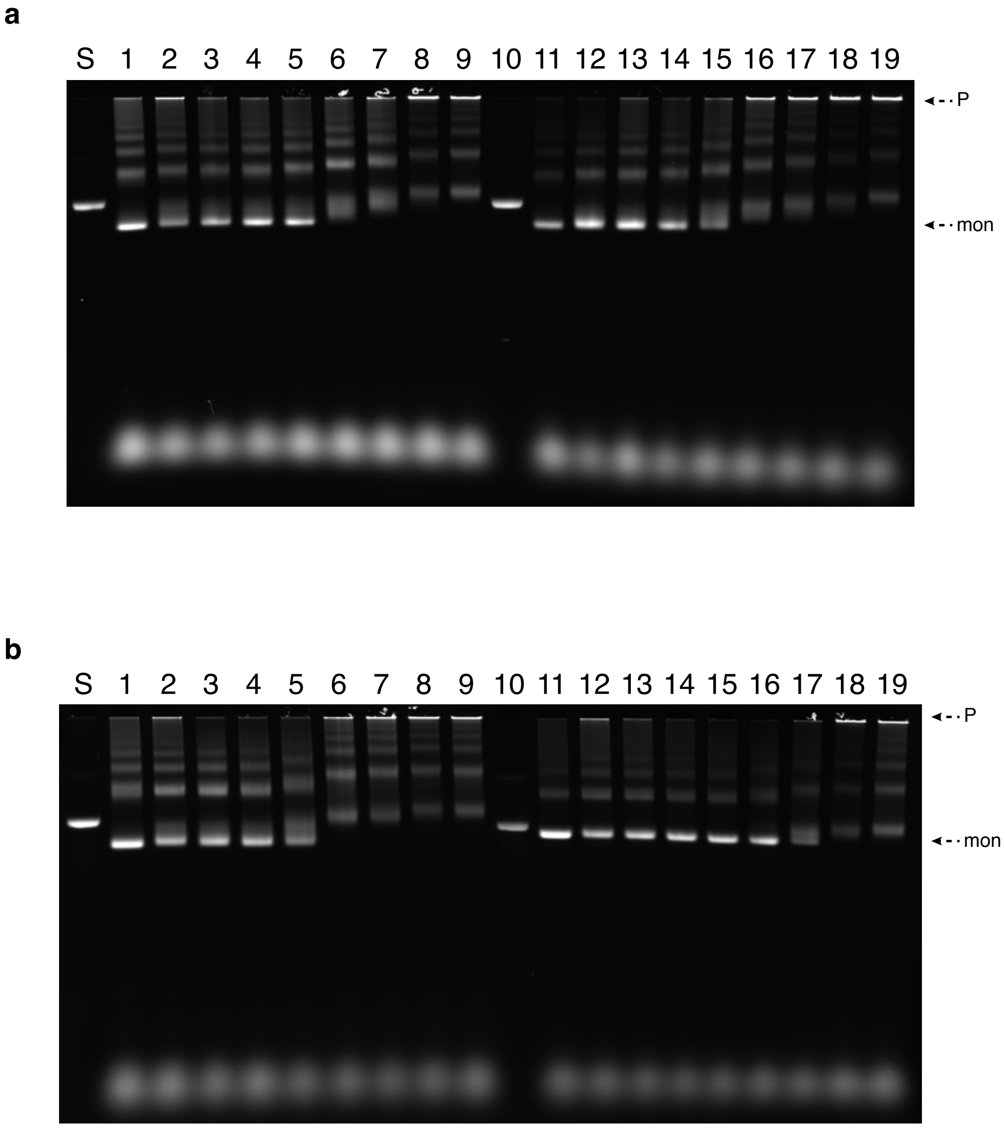

Supplementary Figure 4 | Temperature folding screens of the monomers. (a) Laser-scanned photograph of a 2% agarose gel with temperature folding screens of stator unit 1 (1-9) and stator unit 2 (10-18). **(b)** Laser-scanned photograph of a 2% agarose gel with temperature folding screens of stator unit 3 (1-9) and camshaft (10-18). S1: p8064 scaffold; S2: p7560 scaffold. Lanes 1, 10: 60°C-44°C; lanes 2, 11: 50°C-47°C; lanes 3, 12: 52°C-49°C; lanes 4, 13: 54°C-51°C; lanes 5, 14: 56°C-53°C; lanes 6, 15: 58°C-55°C; lanes 7, 16: 60°C-57°C; lanes 8, 17: 62°C-59°C; lanes 9, 18: 64°C-61°C. P: pockets; mon: monomers.

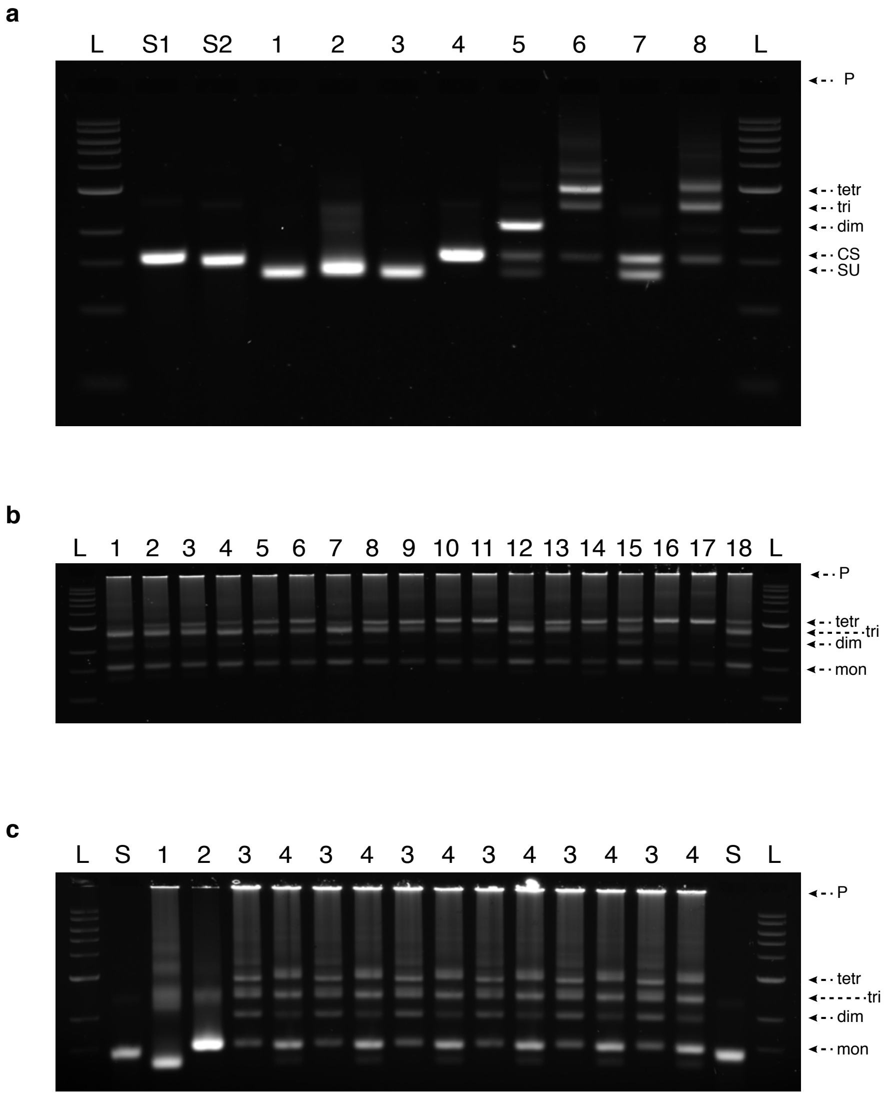

Supplementary Figure 5 | Assembly of the complex. (a) Assembling the complex. Laser-scanned photograph of a 2% agarose gel on which the following samples were electrophoresed: L: ladder; S1: p8064 scaffold; S2: p7560 scaffold; lane 1: stator unit 1; lane 2: stator unit 2; lane 3: stator unit 3; lane 4: camshaft; lane 5: stator unit 1 and camshaft dimers; lane 6: tetrameric complex; lane 7: stator unit 1 and camshaft dimers with invader strands added; lane 8: tetrameric complex with invader strands added. **(b)** Assembly screen for the complex with the camshaft bound to different stator units. Laser-scanned photograph of a 2% agarose gel on which the following samples were electrophoresed: L: ladder; lane 1: camshaft bound to stator unit 2, 20 mM $MgCl_2$, 30°C; lane 2: camshaft bound to stator unit 2, 30 mM $MgCl_2$, 30°C; lane 3: camshaft bound to stator unit 2, 40 mM $MgCl_2$, 30°C; lane 4: camshaft bound to stator unit 3, 20 mM $MgCl_2$, 30°C; lane 5: camshaft bound to stator unit 3, 30 mM $MgCl_2$, 30°C; lane 6: camshaft bound to stator unit 3, 40 mM $MgCl_2$, 30°C; lane 7: camshaft bound to stator unit 2, 20 mM $MgCl_2$, 40°C; lane 8: camshaft bound to stator unit 2, 30 mM $MgCl_2$, 40°C; lane 9: camshaft bound to stator unit 2, 40 mM $MgCl_2$, 40°C; lane 10: camshaft bound to stator unit 3, 30 mM $MgCl_2$, 40°C; lane 11: camshaft bound to stator unit 3, 40 mM $MgCl_2$, 40°C; lane 12: camshaft bound to stator unit 2, 20 mM $MgCl_2$, 50°C; lane 13: camshaft bound to stator unit 2, 30 mM $MgCl_2$, 50°C; lane 14: camshaft bound to stator unit 2, 40 mM $MgCl_2$, 50°C; lane 15: camshaft bound to stator unit 3, 20 mM $MgCl_2$, 50°C; lane 16: camshaft bound to stator unit 3, 30 mM $MgCl_2$, 50°C; lane 17: camshaft bound to stator unit 3, 40 mM $MgCl_2$, 50°C; lane 18: camshaft bound to stator unit 3, 20 mM $MgCl_2$, 40°C. **(c)** Difference between the complex when the central camshaft is bound and not bound. Laser-scanned photograph of a 2% agarose gel on which the following samples were electrophoresed: L: ladder; S: p7560 scaffold; lane 1: stator unit 3; lane 2: camshaft; lanes 3: complex with the camshaft bound to stator unit 1; lanes 4: complex with camshaft free to rotate. P: pockets; tetr: tetramers; tri: trimers; dim: dimers; mon: monomers; CS: camshaft; SU: stator units.

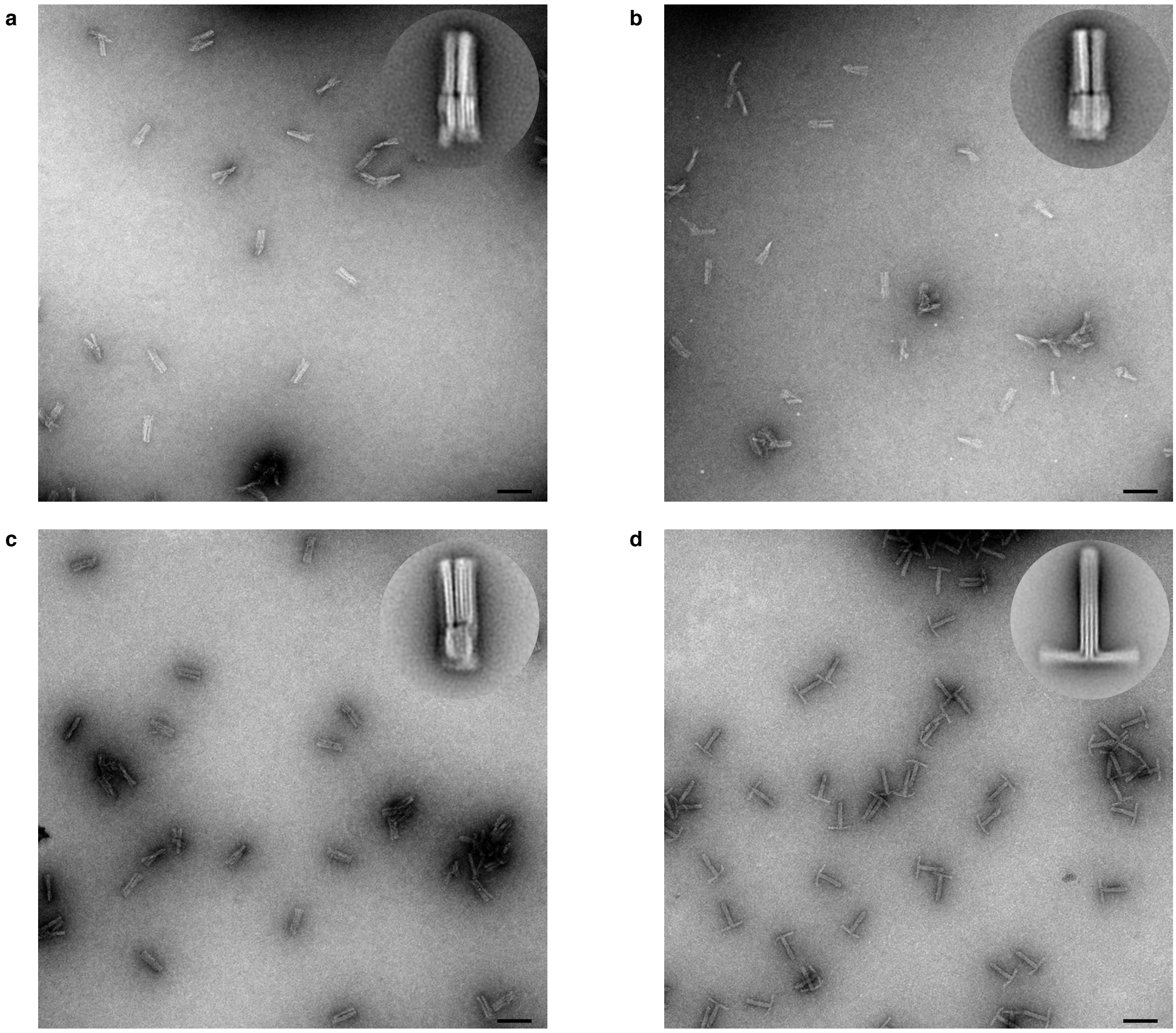

Supplementary Figure 6 | Negative stain electron micrographs of the components of the rotary mechanism. **(a)** Stator unit 1. **(b)** Stator unit 2. **(c)** Stator unit 3. **(d)** Camshaft. Scale bars 100 nm. Insets: 2D class averages of the monomers.

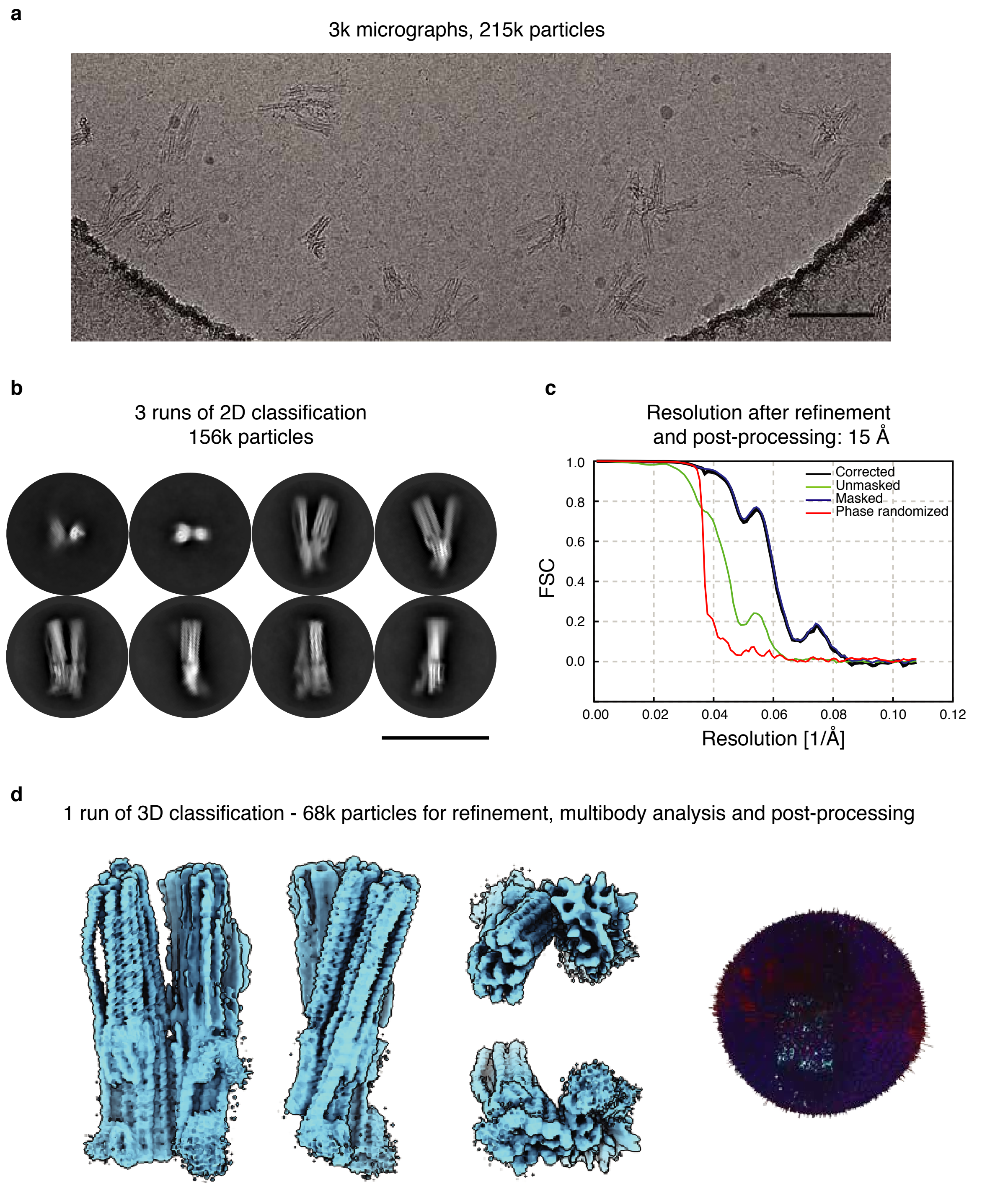

Supplementary Figure 7 | Cryo EM reconstruction of stator unit 1. (a) Exemplary motion-corrected and dose-weighted micrograph. Scale bar 100 nm. **(b)** Exemplary 2D class averages showing the particles in different views. Scale bar 100 nm. **(c)** Fourier Shell Correlation (FSC) of the refined map. **(d)** Composite map from a MultiBody job in different orientations (left) and three-dimensional histogram of the particle orientations (right).

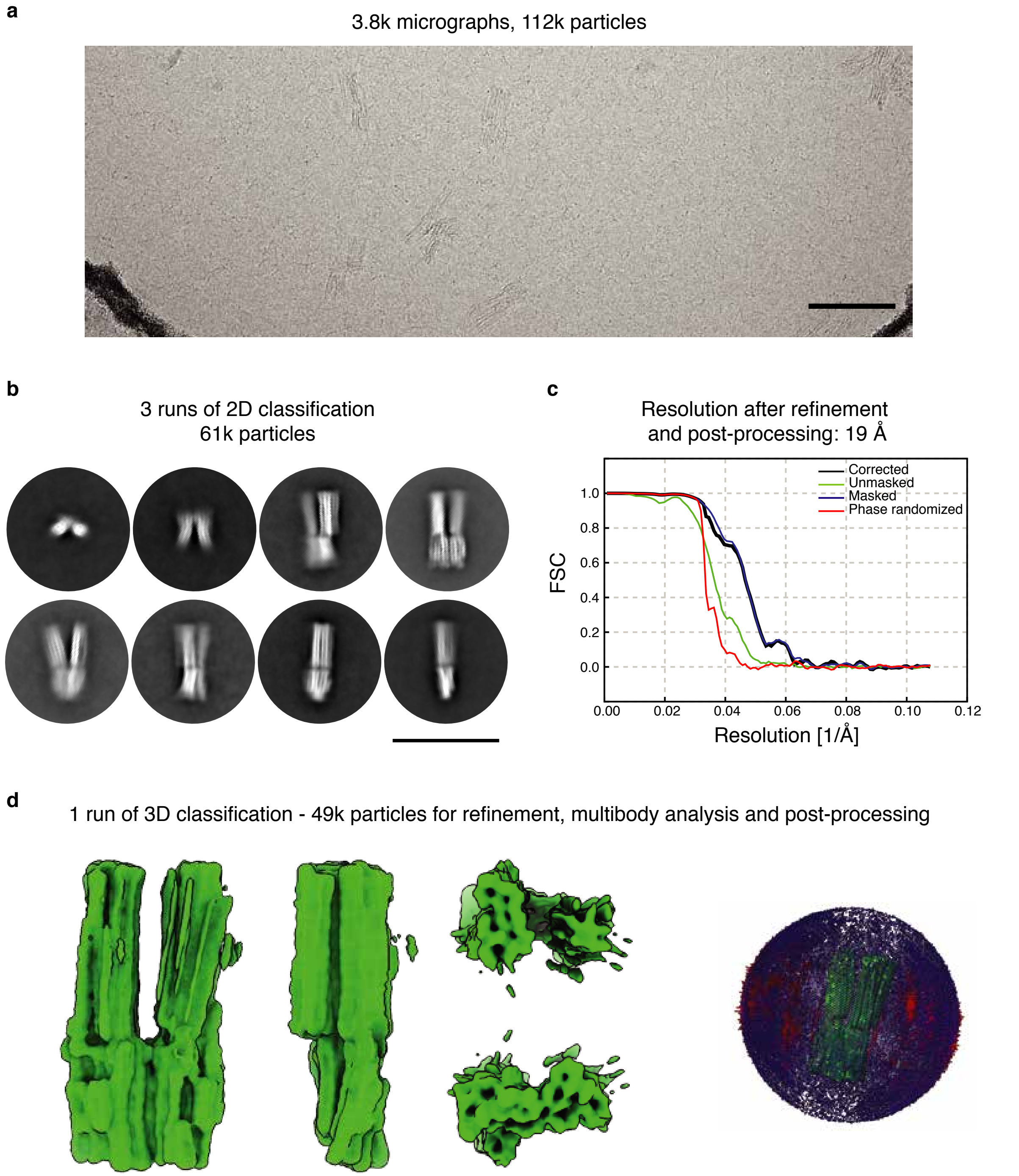

Supplementary Figure 8 | Cryo EM reconstruction of stator unit 2. (a) Exemplary motion-corrected and dose-weighted micrograph. Scale bar 100 nm. **(b)** Exemplary 2D class averages showing the particles in different views. Scale bar 100 nm. **(c)** Fourier Shell Correlation (FSC) of the refined map. **(d)** Composite map from a MultiBody job in different orientations (left) and three-dimensional histogram of the particle orientations (right).

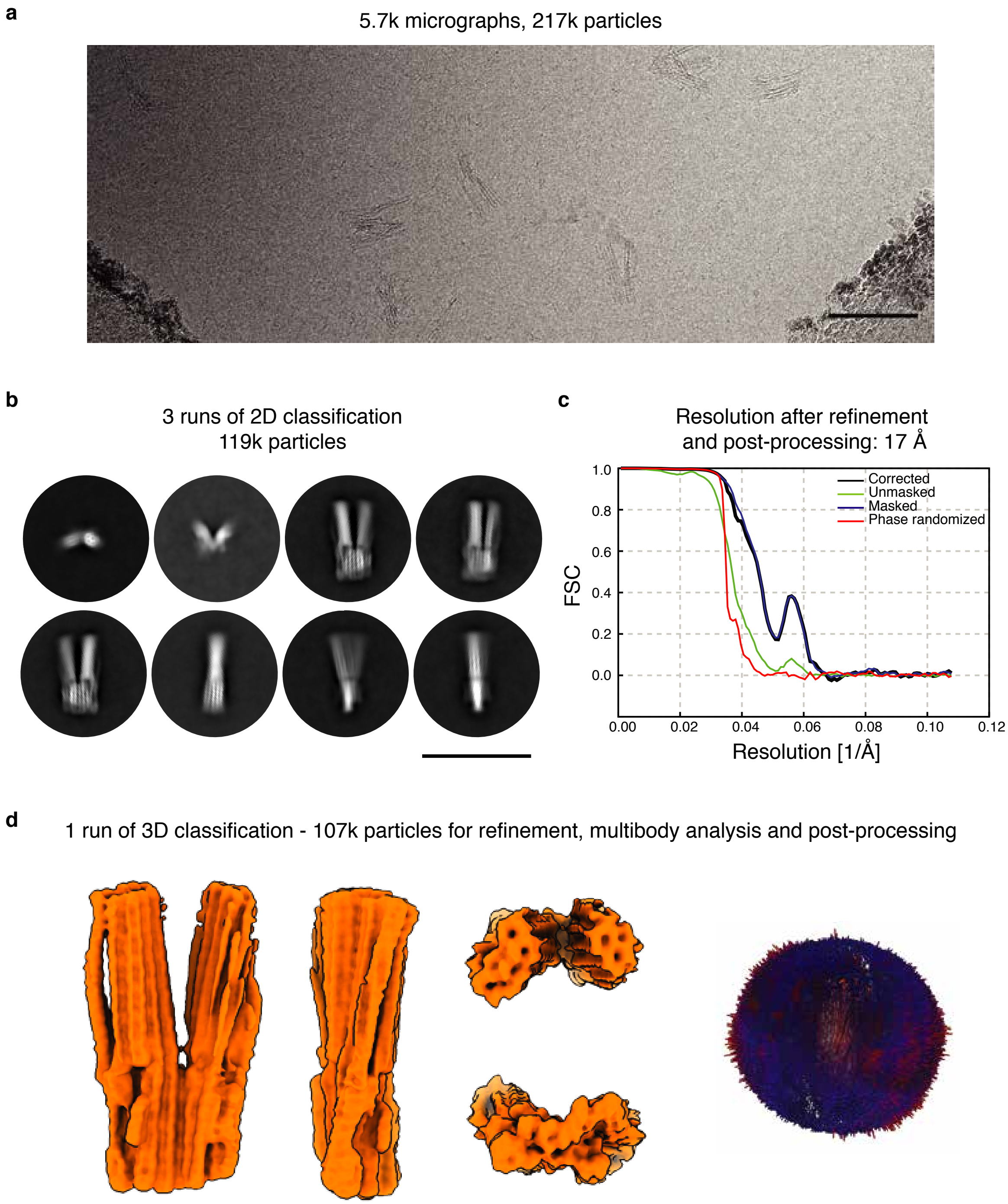

Supplementary Figure 9 | Cryo EM reconstruction of stator unit 3. (a) Exemplary motion-corrected and dose-weighted micrograph. Scale bar 100 nm. **(b)** Exemplary 2D class averages showing the particles in different views. Scale bar 100 nm. **(c)** Fourier Shell Correlation (FSC) of the refined map. **(d)** Composite map from a MultiBody job in different orientations (left) and three-dimensional histogram of the particle orientations (right).

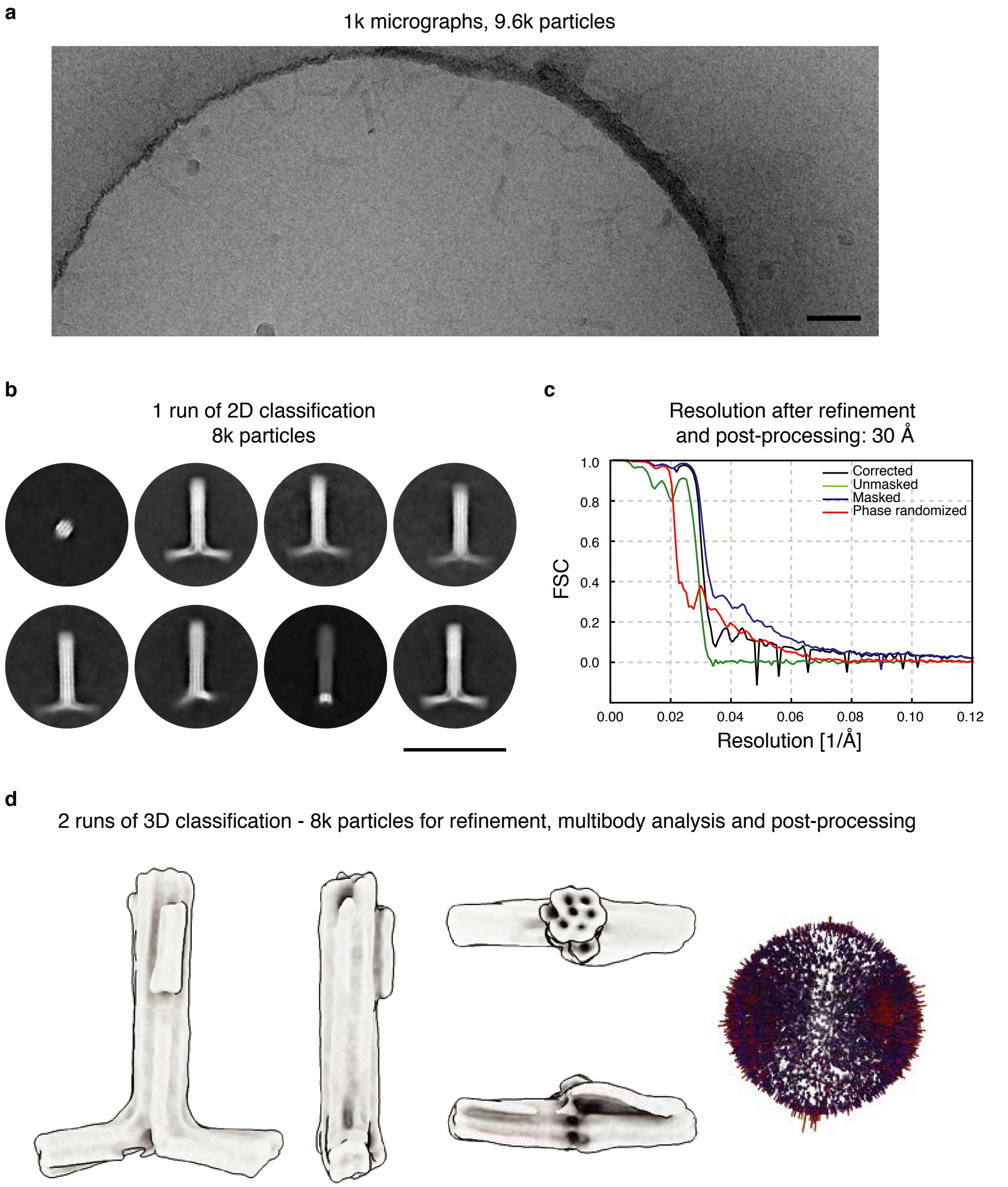

Supplementary Figure 10 | Cryo EM reconstruction of the camshaft. (a) Exemplary micrograph. Scale bar 100 nm. **(b)** Exemplary 2D class averages showing the particles in different views. Scale bar 100 nm. **(c)** Fourier Shell Correlation (FSC) of the refined map. **(d)** Post-processed map in different orientations (left) and three-dimensional histogram of the particle orientations (right).

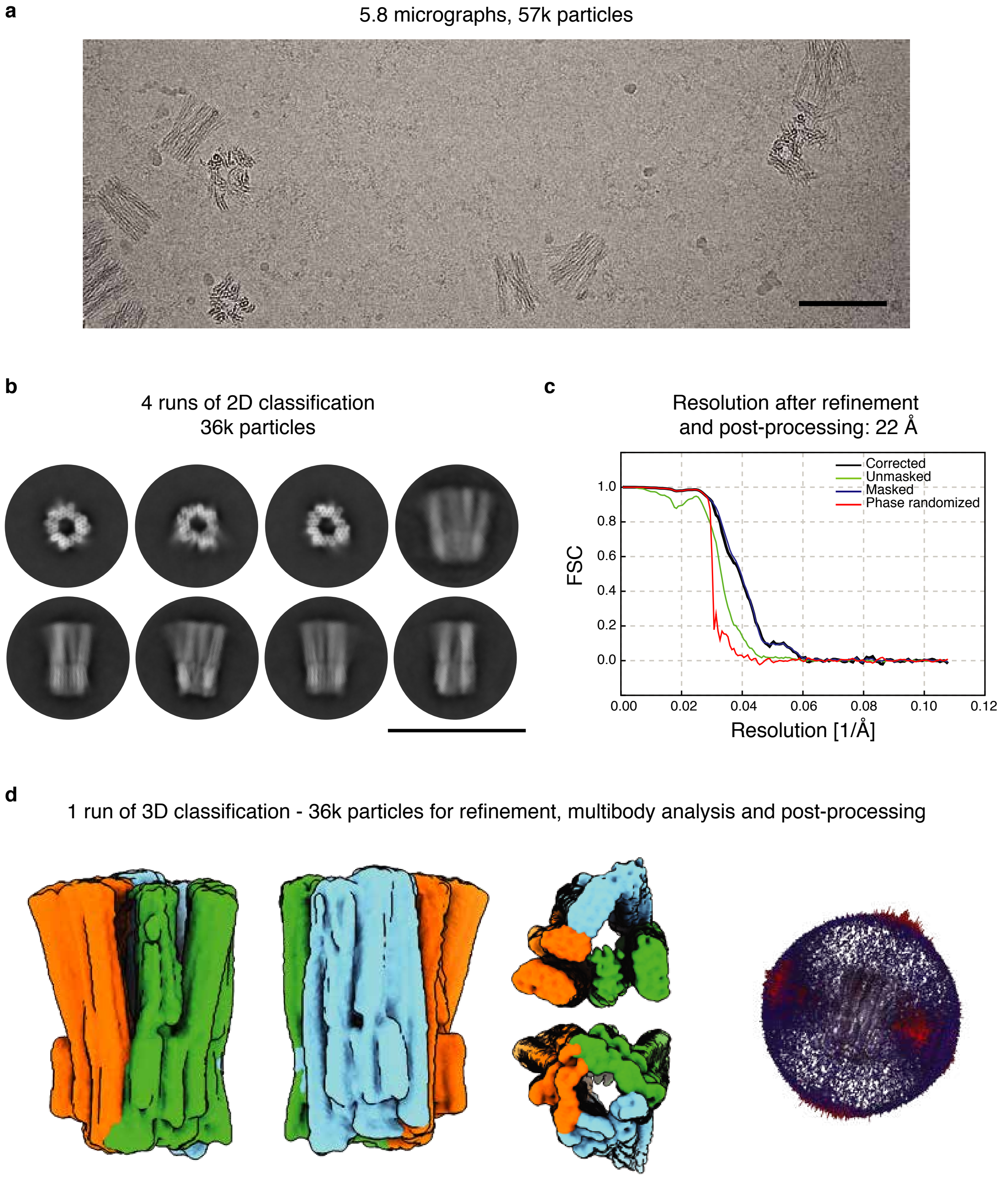

Supplementary Figure 11 | Cryo EM reconstruction of empty stator. (a) Exemplary motion-corrected and dose-weighted micrograph. Scale bar 100 nm. **(b)** Exemplary 2D class averages showing the particles in different views. Scale bar 100 nm. **(c)** Fourier Shell Correlation (FSC) of the refined map. **(d)** Composite map from a MultiBody job in different orientations (left) and three-dimensional histogram of the particle orientations (right).

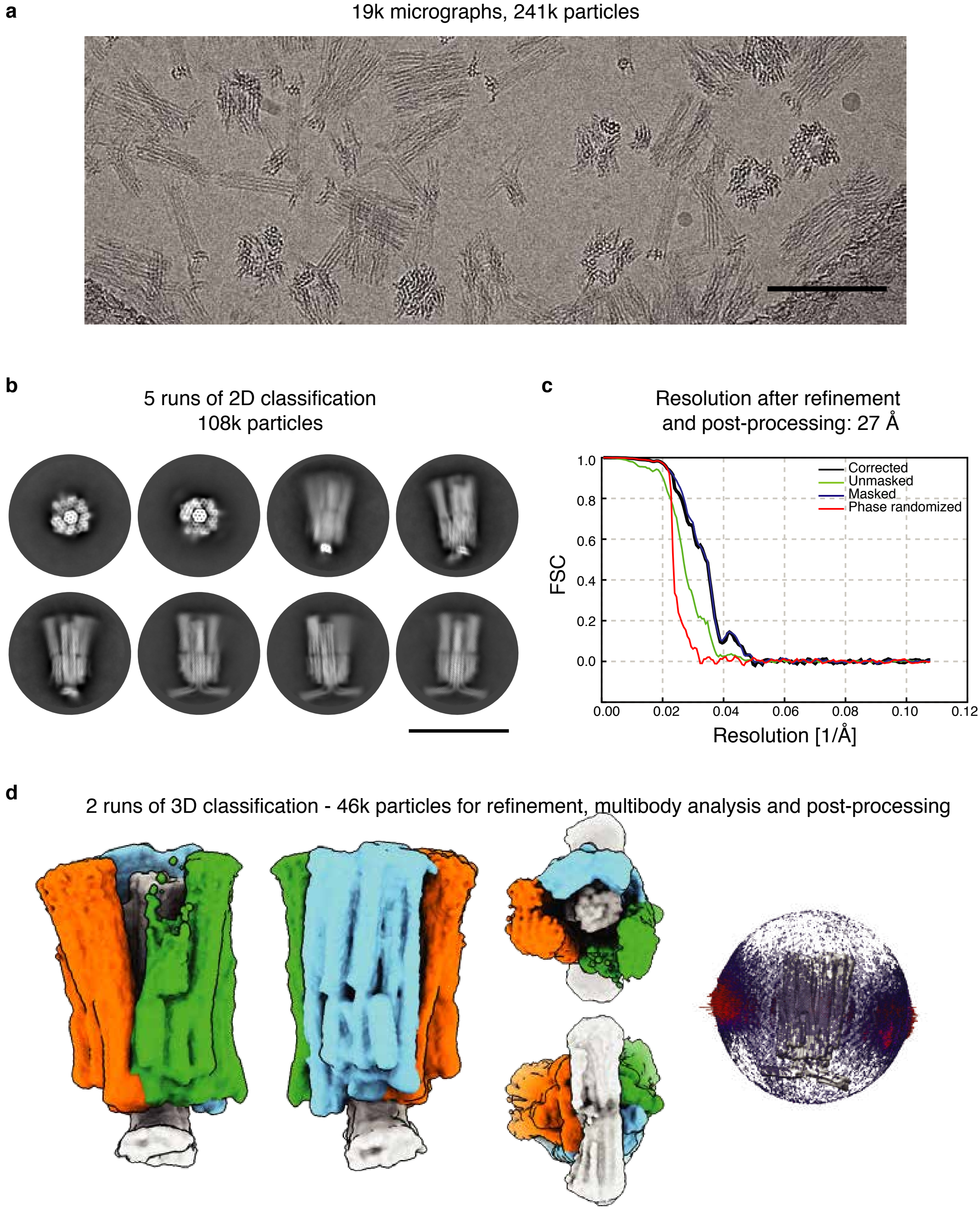

Supplementary Figure 12 | Cryo EM reconstruction of the rotary mechanism with the camshaft bound to stator unit 1. (a) Exemplary motion-corrected and dose-weighted micrograph. Scale bar 100 nm. **(b)** Exemplary 2D class averages showing the particles in different views. Scale bar 100 nm. **(c)** Fourier Shell Correlation (FSC) of the refined map. **(d)** Composite map from a MultiBody job in different orientations (left) and three-dimensional histogram of the particle orientations (right).

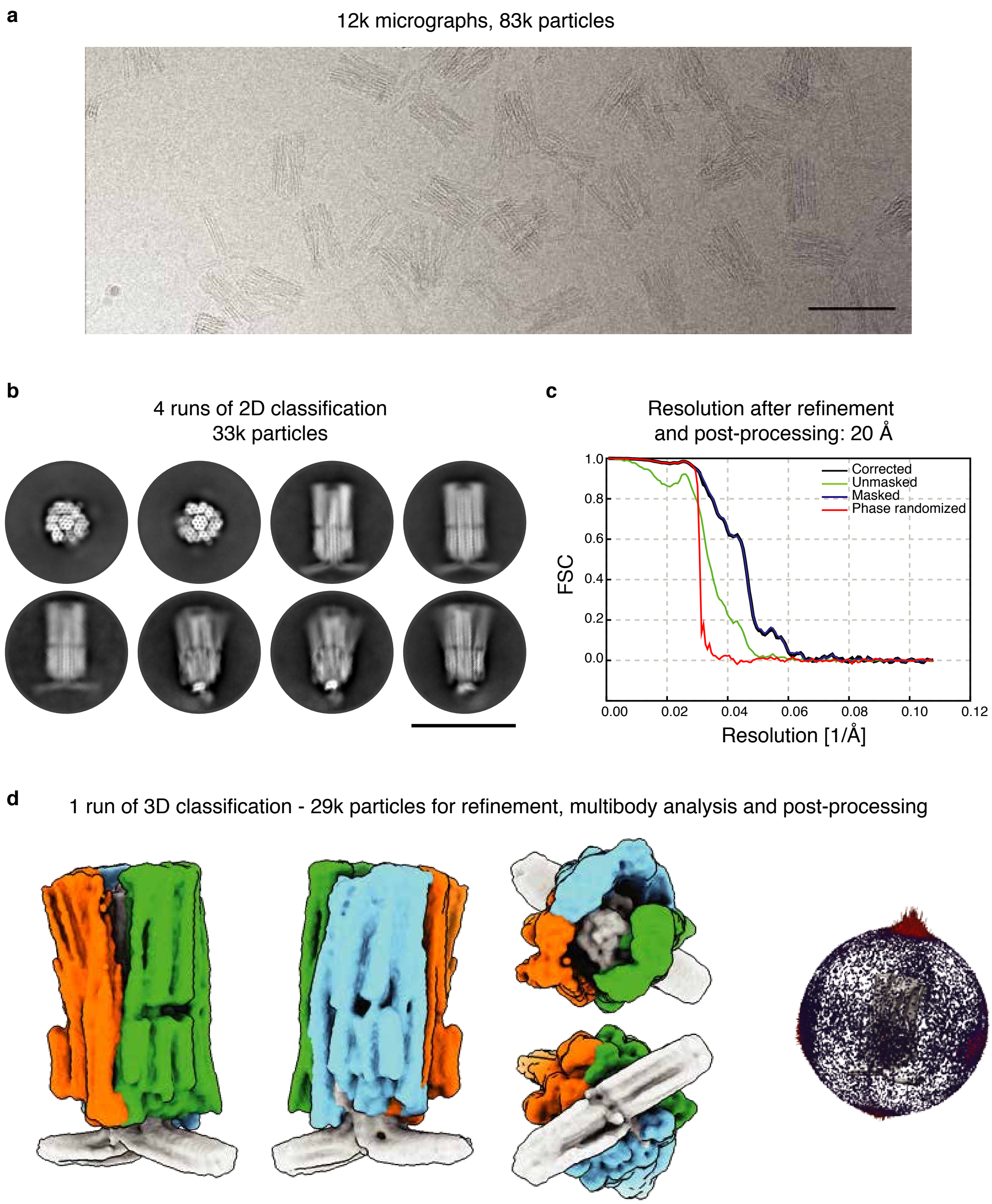

Supplementary Figure 13 | Cryo EM reconstruction of the rotary complex with the camshaft bound to stator unit 2. (a) Exemplary motion-corrected and dose-weighted micrograph. Scale bar 100 nm. **(b)** Exemplary 2D class averages showing the particles in different views. Scale bar 100 nm. **(c)** Fourier Shell Correlation (FSC) of the refined map. **(d)** Composite map from a MultiBody job in different orientations (left) and three-dimensional histogram of the particle orientations (right).

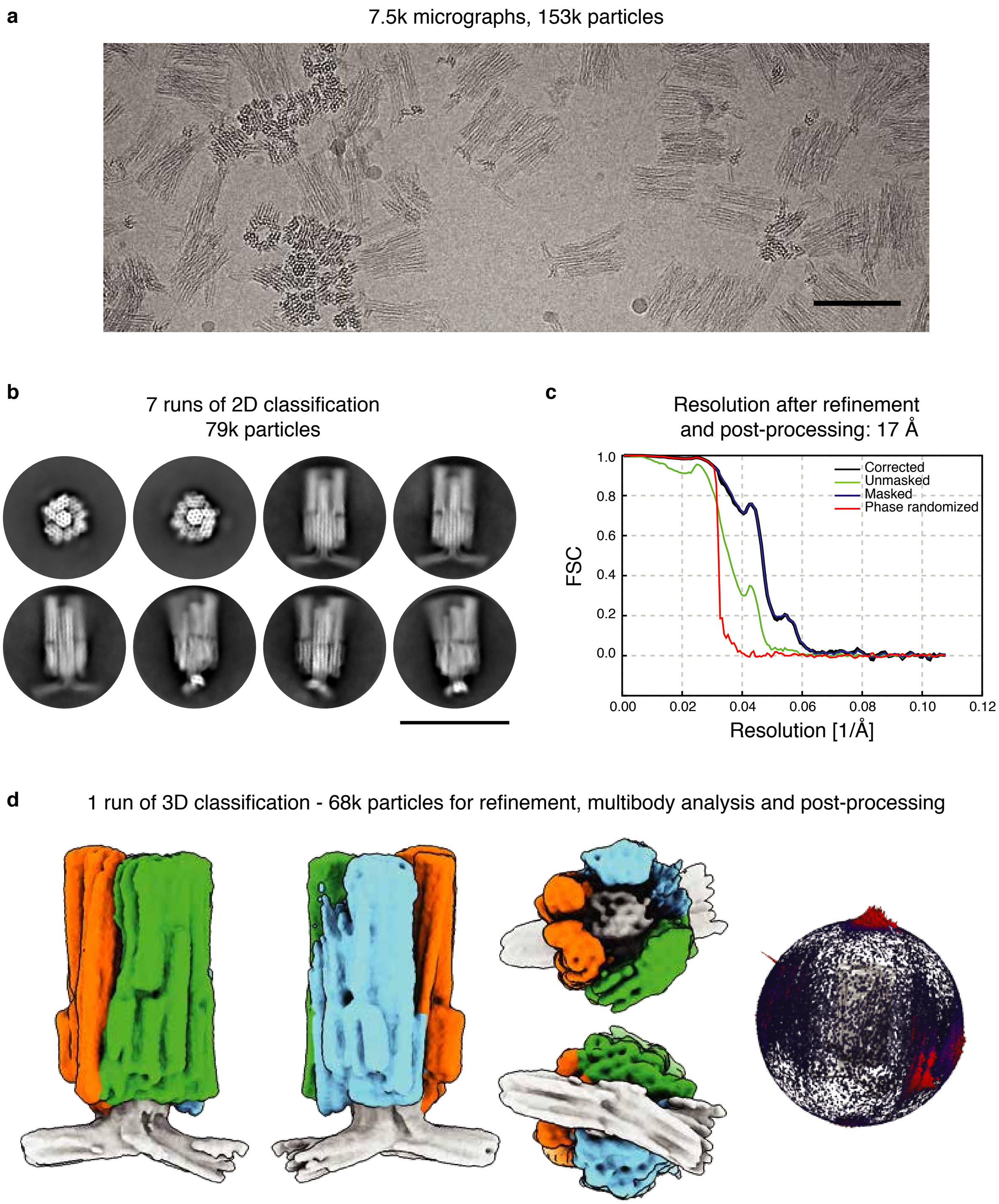

Supplementary Figure 14 Cryo EM reconstruction of the rotary complex with the camshaft bound to stator unit 3. (a) Exemplary motion-corrected and dose-weighted micrograph. Scale bar 100 nm. **(b)** Exemplary 2D class averages showing the particles in different views. Scale bar 100 nm. **(c)** Fourier Shell Correlation (FSC) of the refined map. **(d)** Composite map from a MultiBody job in different orientations (left) and three-dimensional histogram of the particle orientations (right).

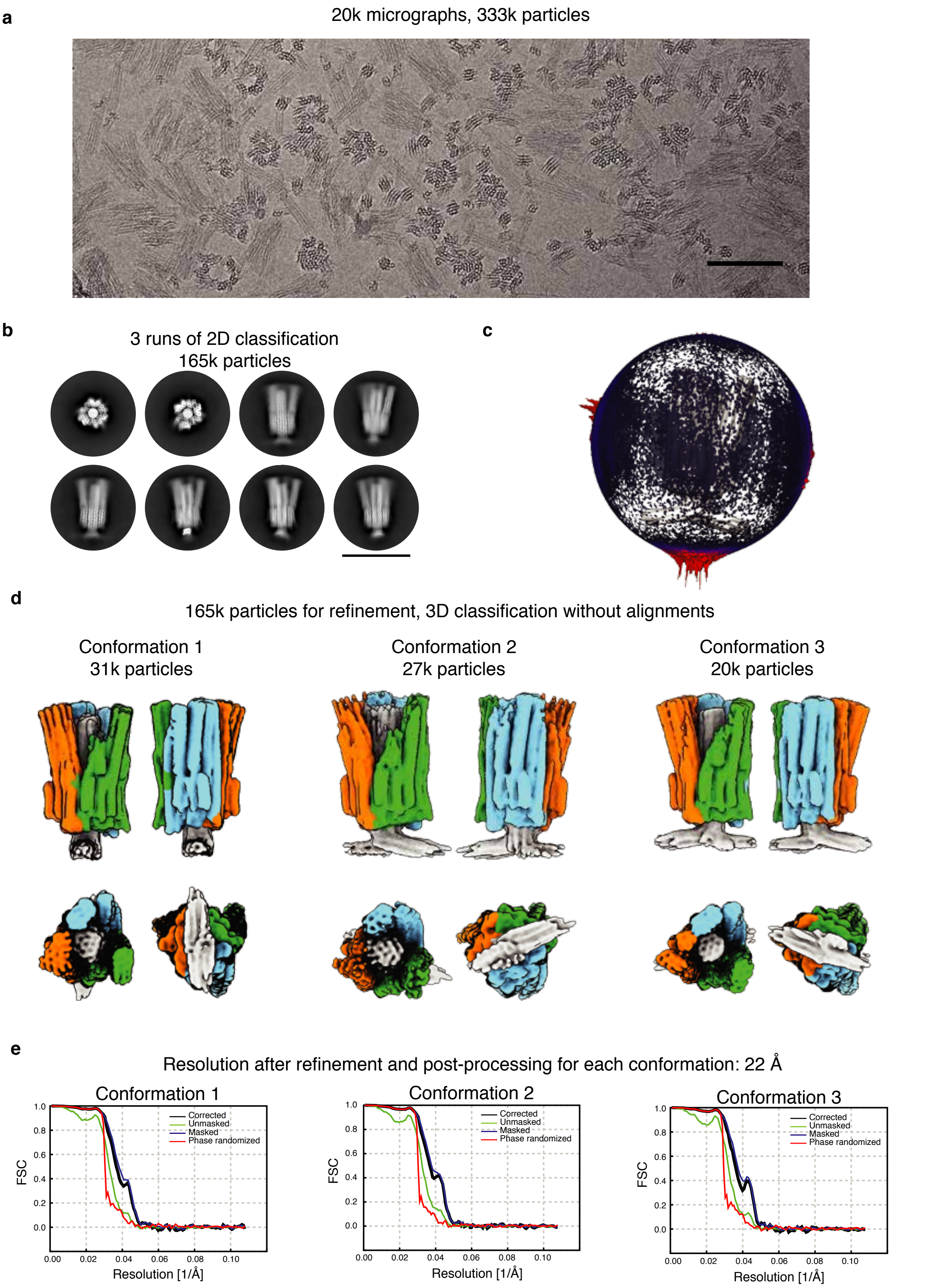

Supplementary Figure 15 | Cryo EM reconstruction of the rotary complex with the camshaft free to rotate. (a) Exemplary motion-corrected and dose-weighted micrograph. Scale bar 100 nm. **(b)** Exemplary 2D class averages showing the particles in different views. Scale bar 100 nm. **(c)** Three-dimensional histogram of the particle orientations. **(d)** Composite map from a MultiBody job in different orientations for the three conformations found in the sample. **(e)** Fourier Shell Correlation (FSC) of the refined maps.

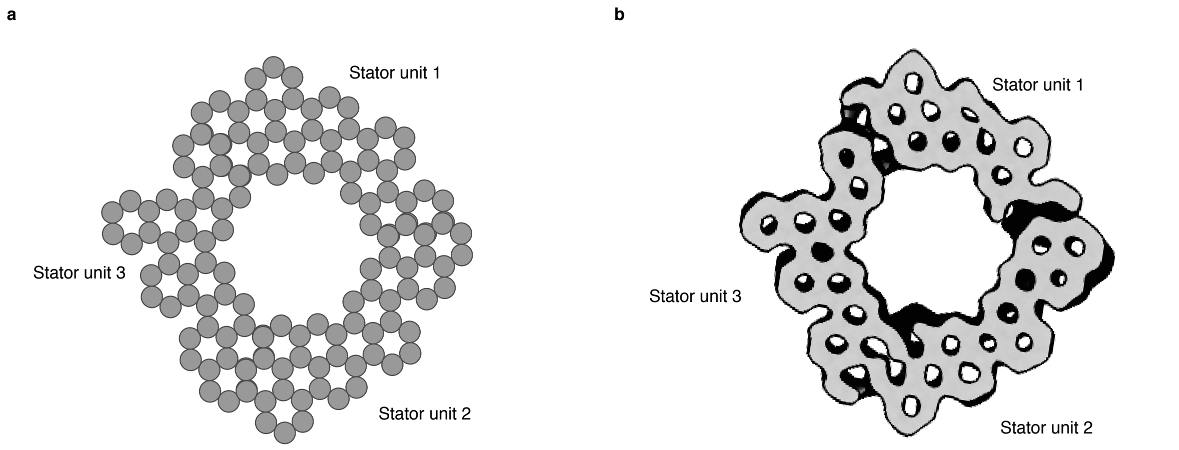

Supplementary Figure 16 | Stator cross section. (a) Designed stator top view cross section, where each circle represents a DNA double helix. **(b)** Slice through a 3D reconstruction of the stator (experimental data) showing the stator cross section.

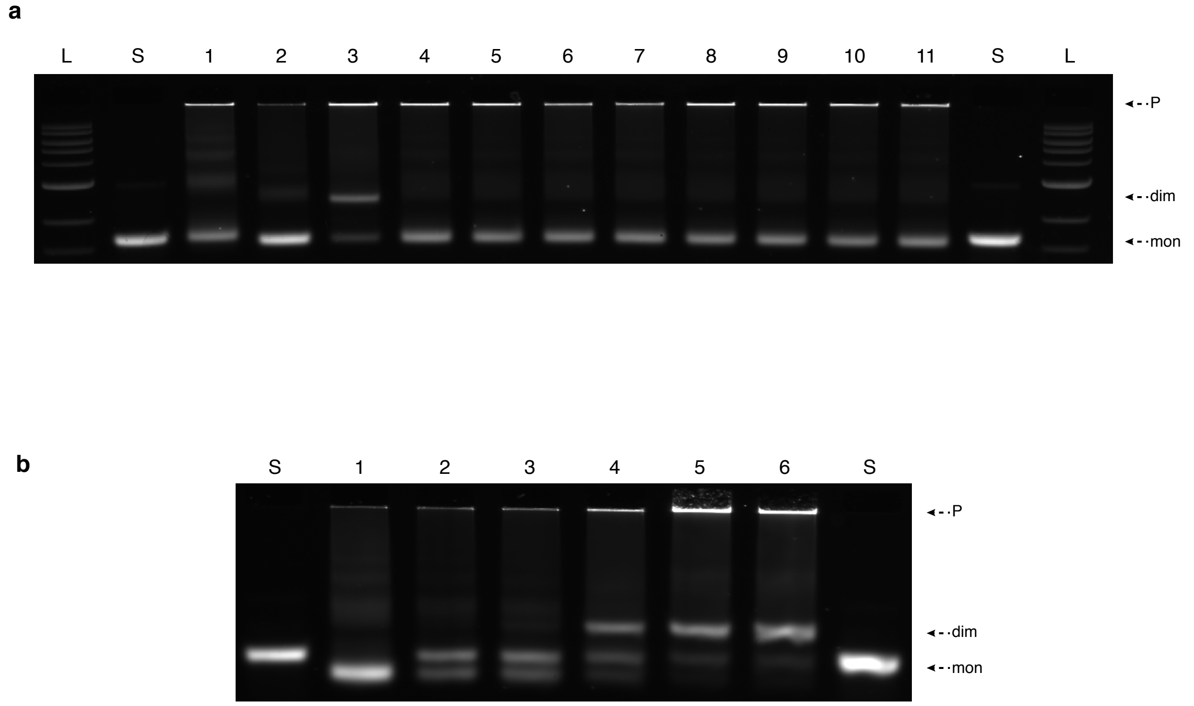

Supplementary Figure 17 | Camshaft release via toehold mediated strand displacement. (a) Incubation time screen of invader. Laser-scanned photograph of a 2% agarose gel on which the following samples were electrophoresed: L: ladder; S: p8064 scaffold; lane 1: stator unit 1; lane 2: camshaft; lane 3: stator unit 1 + camshaft dimers; lane 4-11: stator unit 1 + camshaft dimers to which the invader strands were added in 2x excess for different incubation times; lane 4: 0 min; lane 5: 1 min; lane 6: 5 min; lane 7: 10 min; lane 8: 15 min; lane 9: 20 min; lane 10: 25 min; lane 11: 30 min. **(b)** Sub-stoichiometric excess screen of invader strands. Laser-scanned photograph of a 2% agarose gel on which the following samples were electrophoresed: S: p8064 scaffold; lane 1: stator unit 1; lane 2-5: stator unit 1 + camshaft dimers to which the invader strands were added in different excess; lane 2: 2x excess; lane 3: 1x excess; lane 4: 0.4x excess; lane 5: 0.2x excess; lane 6: stator unit 1 + camshaft dimers. P: pockets; dim: dimers; mon: monomers.

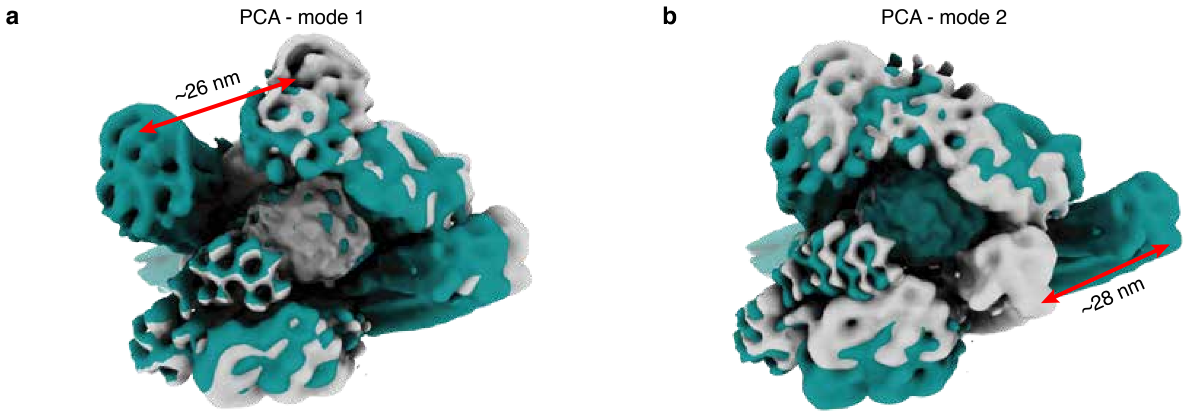

Supplementary Figure 18 | Principal component analysis of the pawls. (a) The two 3D structures (gray and blue) represent the extreme movements of the pawls found in one of the eigenmodes. **(b)** Same as (A) but for another eigenmode. Red arrows: biggest pawl displacements. The input data for this analysis were all the particles in the three 3D classes that showed a different conformation of the camshaft (see final maps in Supplementary Figure 15d). Starting from these maps, 10 rigid bodies were defined: the camshaft, the 6 pawls (2 per each stator unit) and the 3 bearings (1 per each stator unit). With MultiBody and principle component analysis two main motions of the pawls could be identified. The two extremes for each motion were overlapped (in gray and blue, respectively). We identified one helix in each pawl and measured the distance to the same helix in the other extreme conformation. The distances were calculated using UCSF Chimera. Some pawls are subjected to bigger motions (~26/28 nm) while for other pawls the distance difference is around 2 nm.

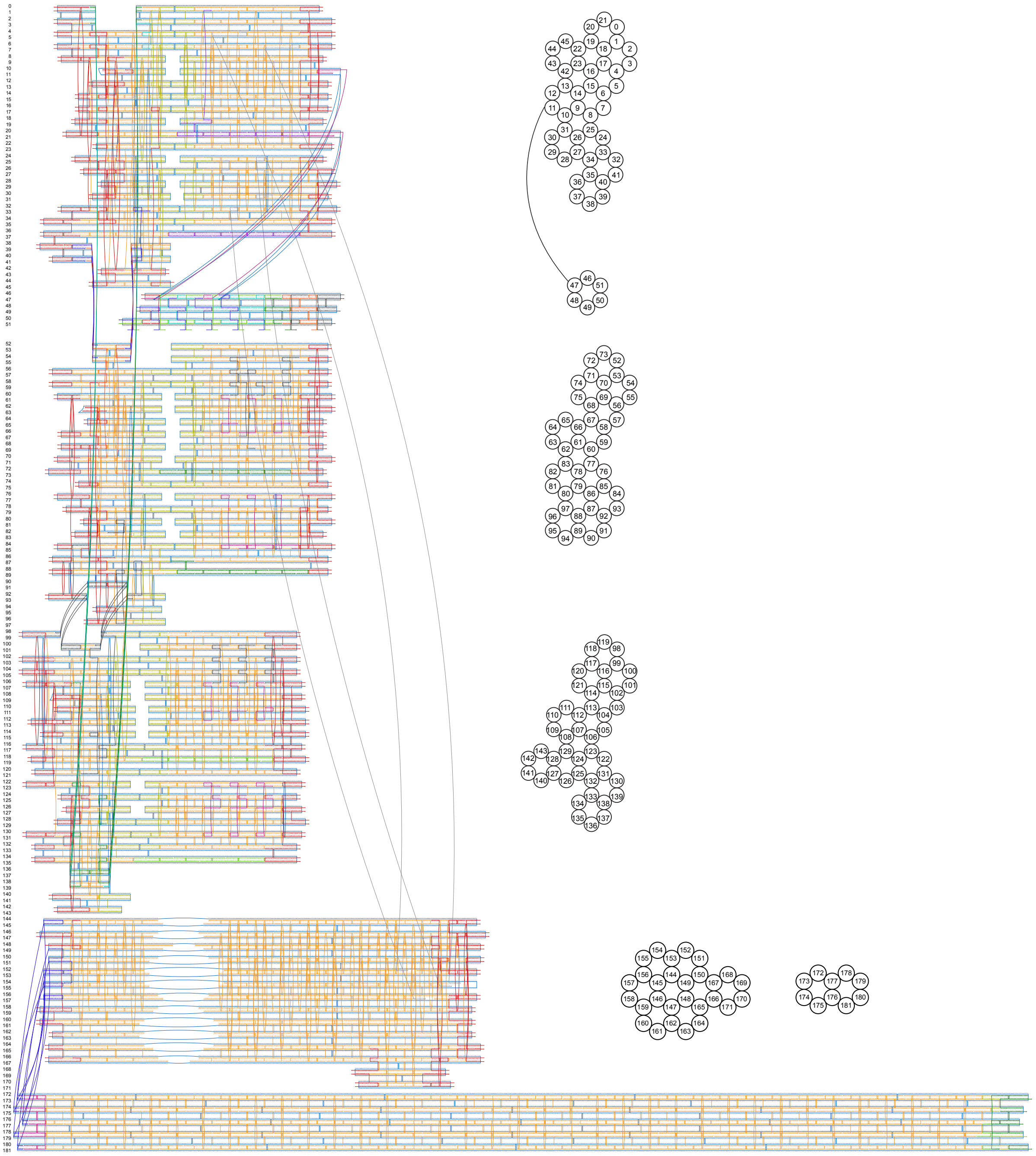

Supplementary Figure 19 | CaDNAno (*1*) design diagram and bottom view cross sections of the rotary mechanism with modifications for TIRFM analysis.

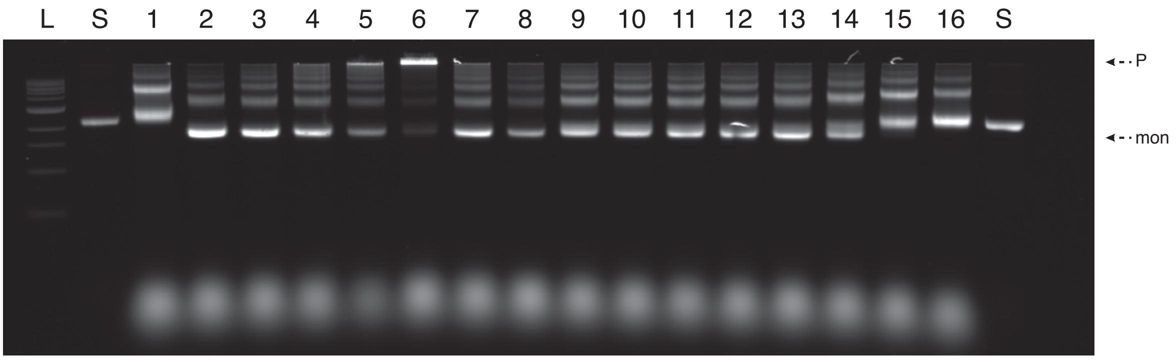

Supplementary Figure 20 | Folding screen of stator unit 1 with modifications for anchoring to a TIRFM glass slide. Laser-scanned photograph of a 2% agarose gel on which the following samples were electrophoresed: L: ladder; S: p9072 scaffold; lane 1: 60°C-44°C, 5 mM $MgCl_2$; lane 2: 60°C-44°C, 10 mM $MgCl_2$; lane 3: 60°C-44°C, 15 mM $MgCl_2$; lane 4: 60°C-44°C, 20 mM $MgCl_2$; lane 5: 60°C-44°C, 25 mM $MgCl_2$; lane 6: 60°C-44°C, 30 mM $MgCl_2$; lane 7: 4x staples-to-scaffold excess; lane 8: 10x staples-to-scaffold excess; lane 9: 50°C-47°C, 20 mM $MgCl_2$; lane 10: 52°C-49°C, 20 mM $MgCl_2$; lane 11: 54°C-51°C, 20 mM $MgCl_2$; lane 12: 56°C-53°C, 20 mM $MgCl_2$; lane 13: 58°C-55°C, 20 mM $MgCl_2$; lane 14: 60°C-57°C, 20 mM $MgCl_2$; lane 15: 62°C-59°C; lane 16: 64°C-61°C, 20 mM $MgCl_2$. P: pockets; mon: monomers.

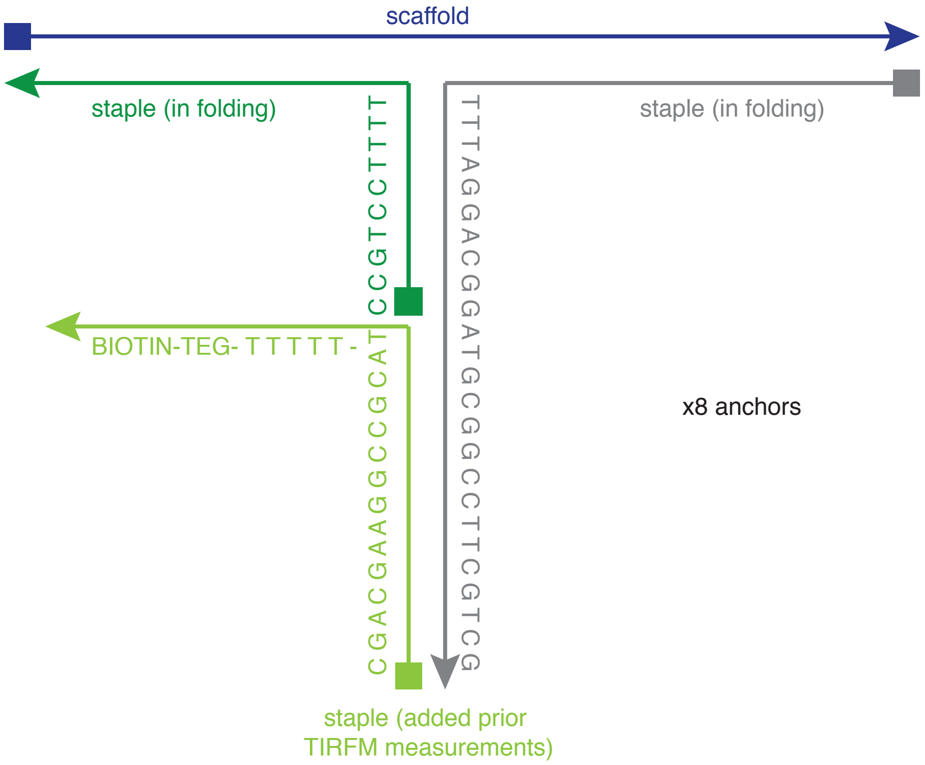

Supplementary Figure 21 | Schematic representation of the oligos used for anchoring the stator unit 1 on a TIRFM glass slide.

a

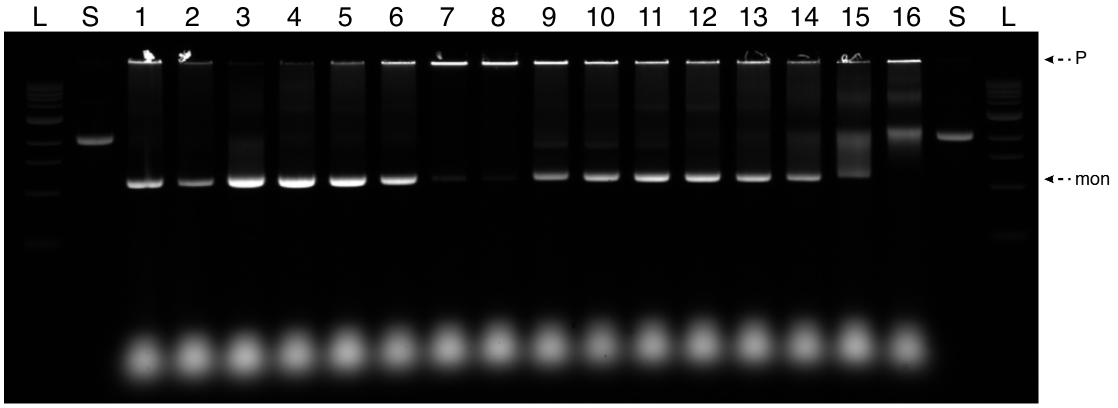

b

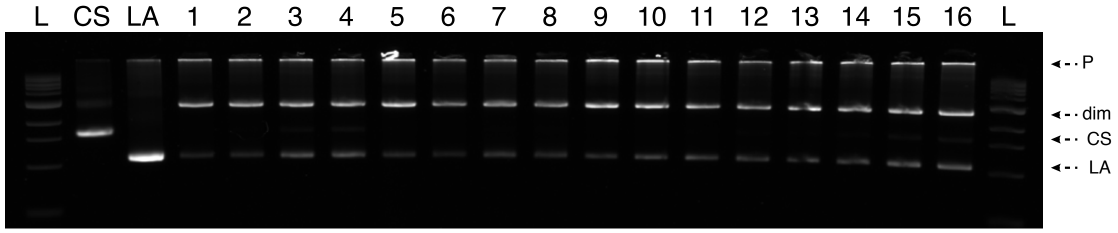

Supplementary Figure 22 | Folding screen for lever arm and dimerization screen to camshaft. (a) Folding screen for the lever arm. Laser-scanned photograph of a 2% agarose gel on which the following samples were electrophoresed: L: ladder; S: p8064 scaffold; lane 1: 4x staples-to-scaffold excess; lane 2: 10x staples-to-scaffold excess; lane 3: 60°C-44°C, 5 mM $MgCl_2$; lane 4: 60°C-44°C, 10 mM $MgCl_2$; lane 5: 60°C-44°C, 15 mM $MgCl_2$; lane 6: 60°C-44°C, 20 mM $MgCl_2$; lane 7: 60°C-44°C, 25 mM $MgCl_2$; lane 8: 60°C-44°C, 30 mM $MgCl_2$; lane 9: 50°C-47°C, 20 mM $MgCl_2$; lane 10: 52°C-49°C, 20 mM $MgCl_2$; lane 11: 54°C-51°C, 20 mM $MgCl_2$; lane 12: 56°C-53°C, 20 mM $MgCl_2$; lane 13: 58°C-55°C, 20 mM $MgCl_2$; lane 14: 60°C-57°C, 20 mM $MgCl_2$; lane 15: 62°C-59°C, 20 mM $MgCl_2$; lane 16: 64°C-61°C, 20 mM $MgCl_2$. **(b)** Dimerization screen between the camshaft and the lever arm. Laser-scanned photograph of a 2% agarose gel on which the following samples were electrophoresed: L: ladder; CS: camshaft; LA: lever arm; lane 1: 20 mM $MgCl_2$, RT; lane 2: 30 mM $MgCl_2$, RT; lane 3: 40 mM $MgCl_2$, RT; lane 4: 50 mM $MgCl_2$, RT; lane 5: 20 mM $MgCl_2$, 30°C; lane 6: 30 mM $MgCl_2$, 30°C; lane 7: 40 mM $MgCl_2$, 30°C; lane 8: 50 mM $MgCl_2$, 30°C; lane 9: 20 mM $MgCl_2$, 40°C; lane 10: 30 mM $MgCl_2$, 40°C; lane 11: 40 mM $MgCl_2$, 40°C; lane 12: 50 mM $MgCl_2$, 40°C; lane 13: 20 mM $MgCl_2$, 50°C; lane 14: 30 mM $MgCl_2$, 50°C; lane 15: 40 mM $MgCl_2$, 50°C; lane 16: 50 mM $MgCl_2$, 50°C. P: pockets; dim: dimers; mon: monomers.

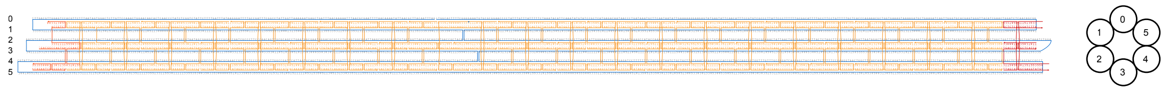

Supplementary Figure 23 | CaDNAno (*1*) design diagram (left) and bottom-view cross section (right) of the prolongation of the 6hb in stator unit 1.

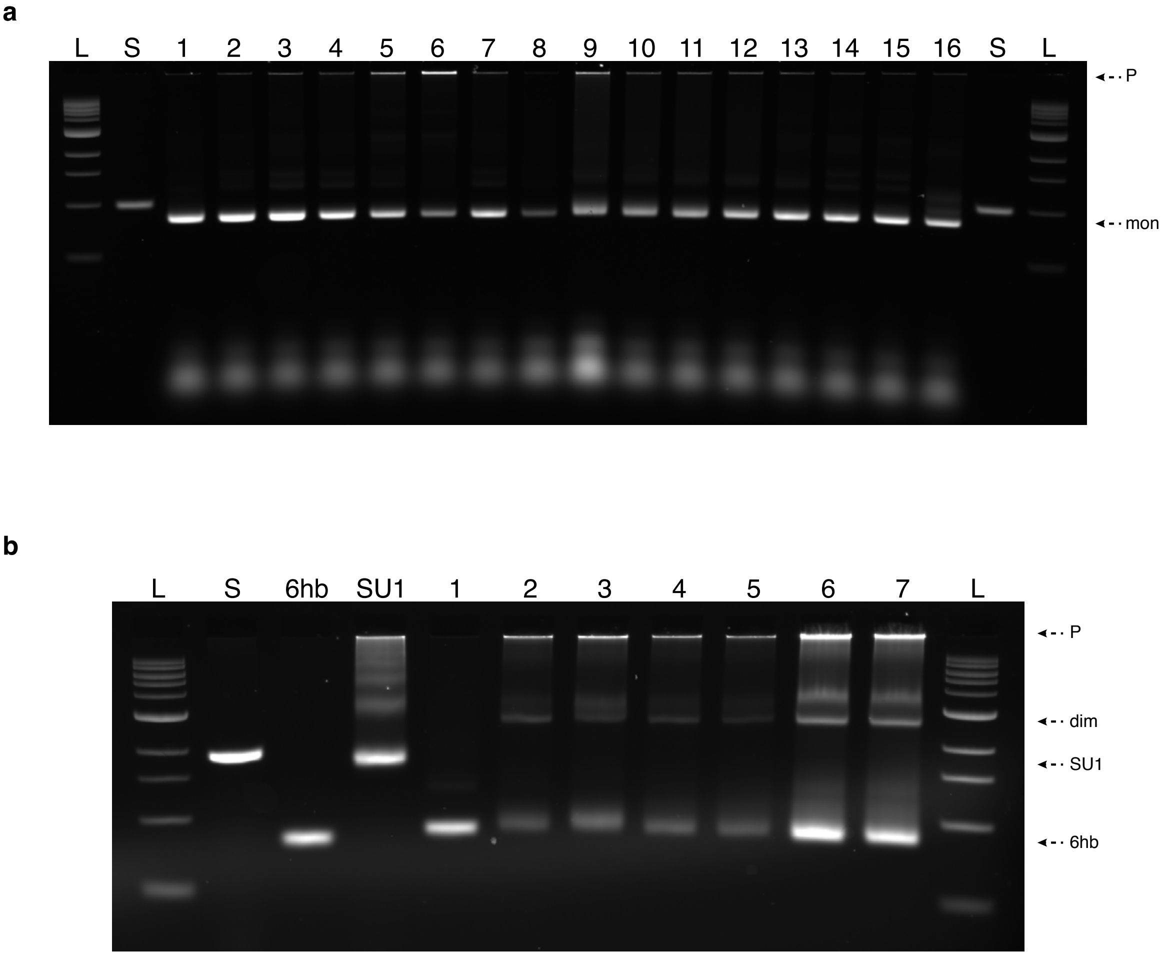

Supplementary Figure 24 | Folding screen for the 6hb and dimerization screen with stator unit 1. (a) Folding screen for the 6hb. Laser-scanned photograph of a 2% agarose gel on which the following samples were electrophoresed: L: ladder; S: p2873 scaffold; lane 1: 60°C-44°C, 5 mM $MgCl_2$; lane 2: 60°C-44°C, 10 mM $MgCl_2$; lane 3: 60°C-44°C, 15 mM $MgCl_2$; lane 4: 60°C-44°C, 20 mM $MgCl_2$; lane 5: 60°C-44°C, 25 mM $MgCl_2$; lane 6: 60°C-44°C, 30 mM $MgCl_2$; lane 7: 4x staples-to-scaffold excess; lane 8: 10x staples-to-scaffold excess; lane 9: 50°C-47°C, 20 mM $MgCl_2$; lane 10: 52°C-49°C, 20 mM $MgCl_2$; lane 11: 54°C-51°C, 20 mM $MgCl_2$; lane 12: 56°C-53°C, 20 mM $MgCl_2$; lane 13: 58°C-55°C, 20 mM $MgCl_2$; lane 14: 60°C-57°C, 20 mM $MgCl_2$; lane 15: 62°C-59°C; lane 16: 64°C-61°C, 20 mM $MgCl_2$. **(b)** Dimerization screen between the stator unit 1 and the 6hb. Laser-scanned photograph of a 2% agarose gel on which the following samples were electrophoresed: L: ladder; S1: p9072 scaffold; S2: p2873 scaffold; SU1: stator unit 1; 6hb: 6 helix-bundle; lane 1: 40 mM $MgCl_2$, 30°C; lane 2: 50 mM $MgCl_2$, 30°C; lane 3: 40 mM $MgCl_2$, 40°C; lane 4: 50 mM $MgCl_2$, 40°C; lane 5: 40 mM $MgCl_2$, 50°C; lane 6: 50 mM $MgCl_2$, 50°C. P: pockets; mon: monomers; dim: dimers.

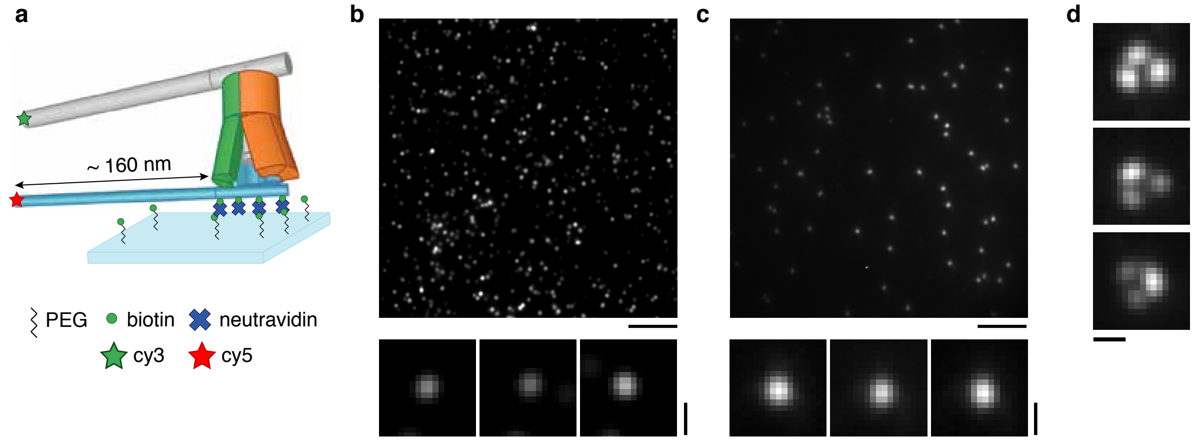

Supplementary Figure 25 | Control measurements on TIRFM. (a) Schematic representation of the complex with the extended pointer. **(b)** Typical field of view image (top, scale bar: 5 μm) and single-particle standard deviation images (bottom, scale bar: 600 nm) in the cy5 channel (stator) for the sample with the extended pointer. **(c)** Typical field of view image (top, scale bar: 5 μm) and single-particle standard deviation images (bottom, scale bar: 600 nm) in the cy3 channel (lever arm) for the sample with the camshaft bound to the stator unit 1. **(d)** Comparison with standard deviation images of particles switching between 3 spots (see Figure 4). Scale bar: 600 nm.

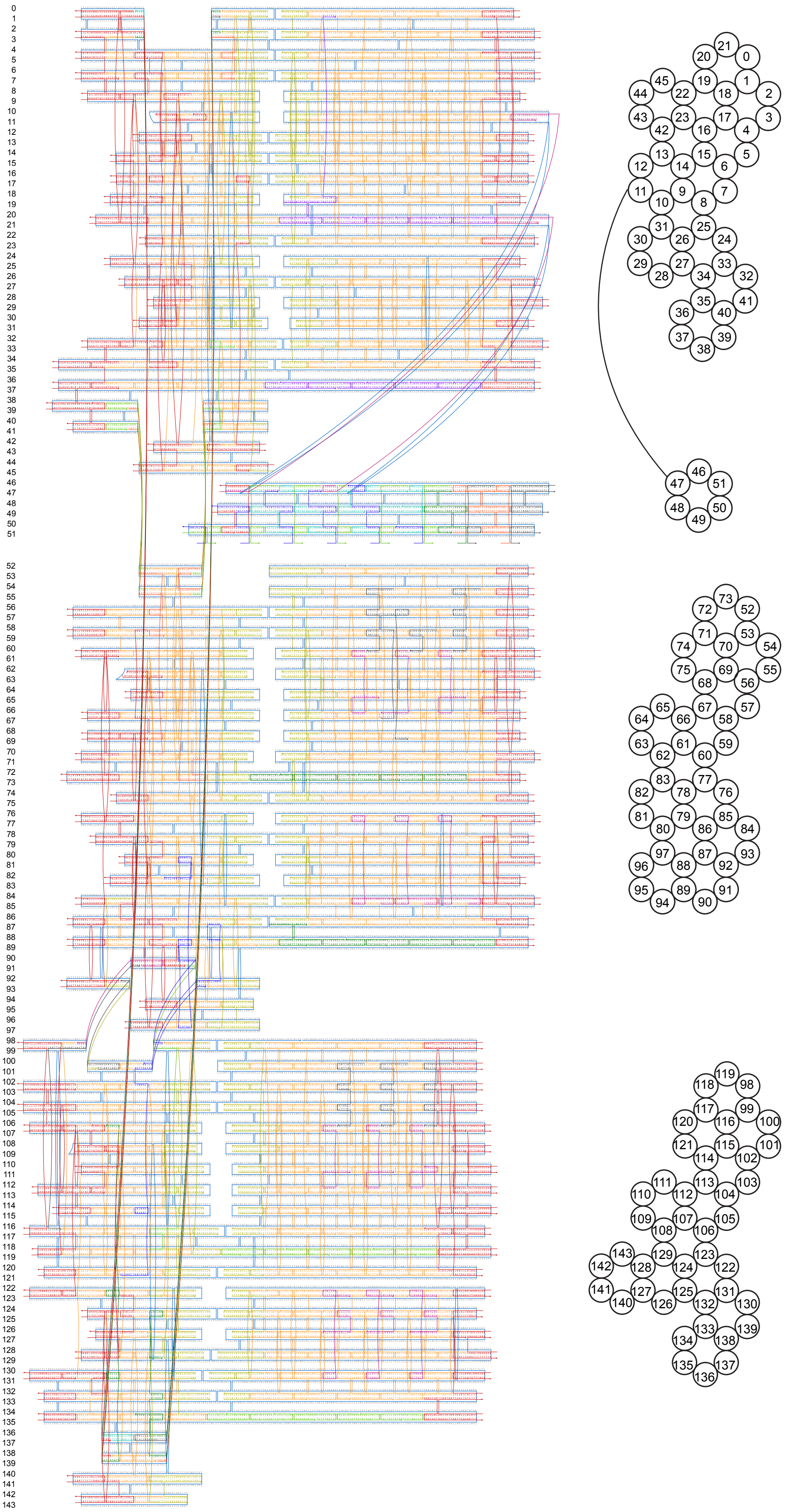

Supplementary Figure 26 | CaDNAno (*1*) design diagram (left) and bottom view cross sections (right) of v2 stator units.

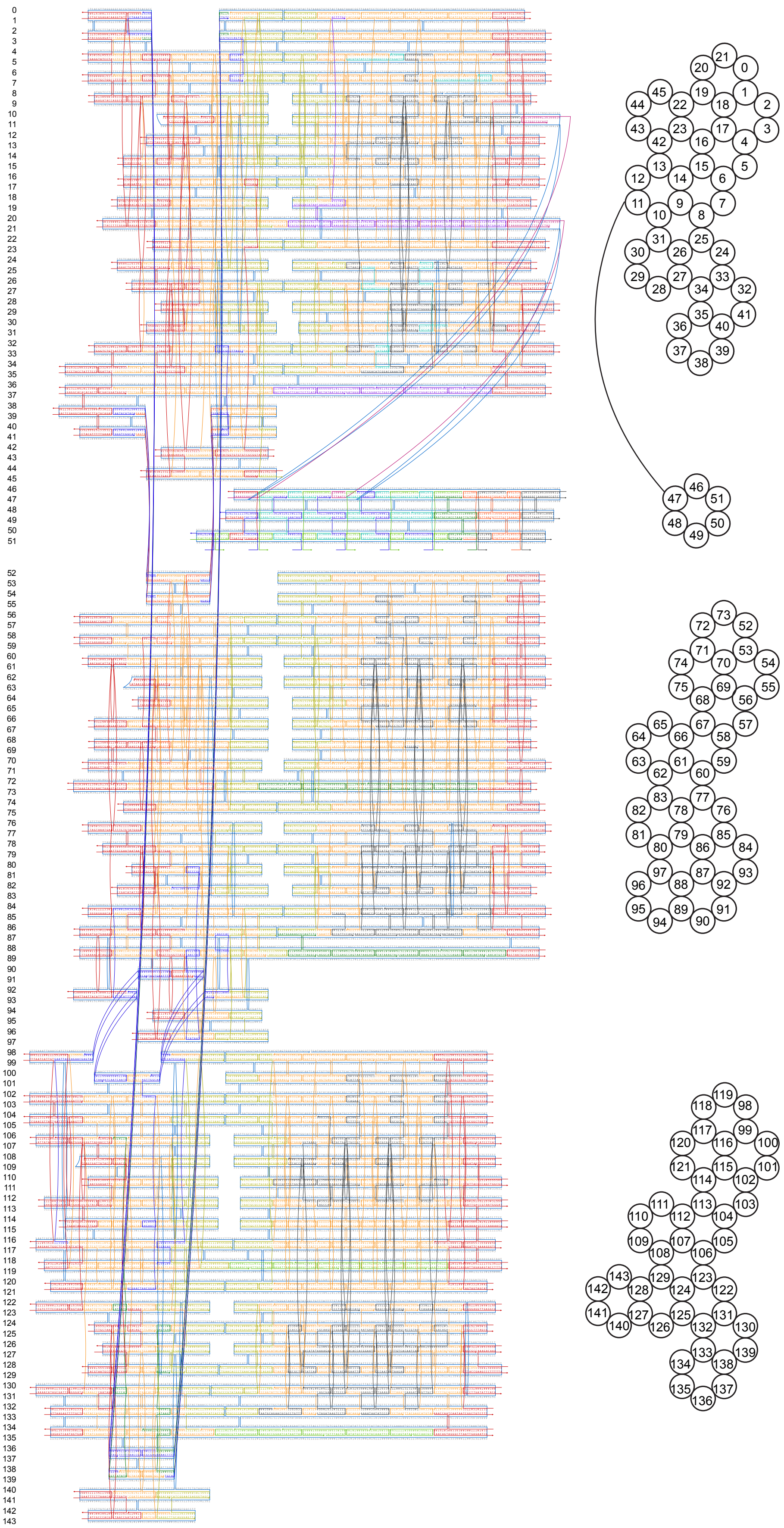

Supplementary Figure 27 | CaDNAno (*1*) design diagram (left) and bottom view cross sections (right) of v3 stator units.

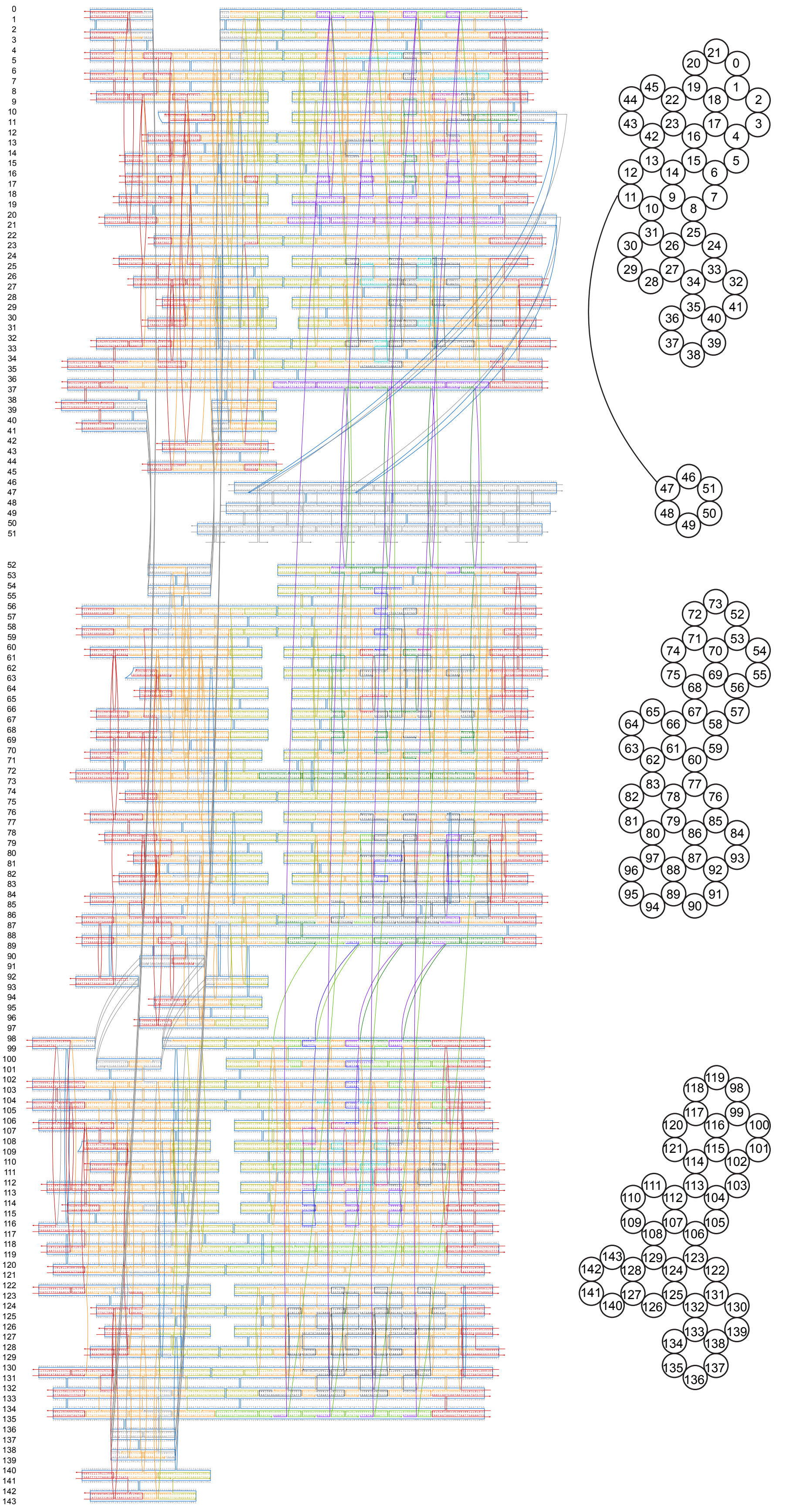

Supplementary Figure 28 | CaDNAno (*1*) design diagram (left) and bottom view cross sections (right) of v4 stator units.

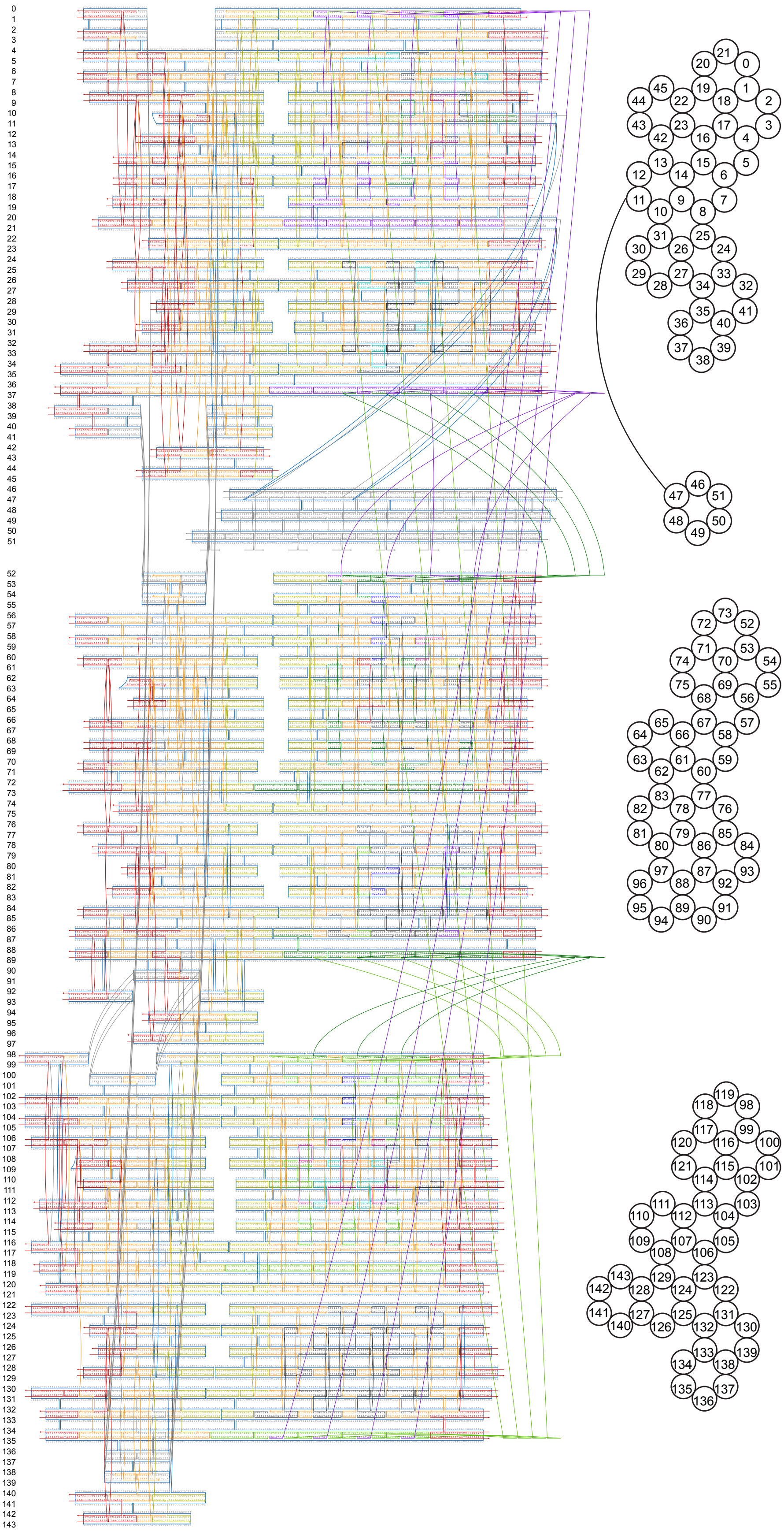

Supplementary Figure 29 | CaDNAno (*1*) design diagram (left) and bottom view cross sections (right) of v5 stator units. The spacer oligos between the stator units are 25 Ts long.

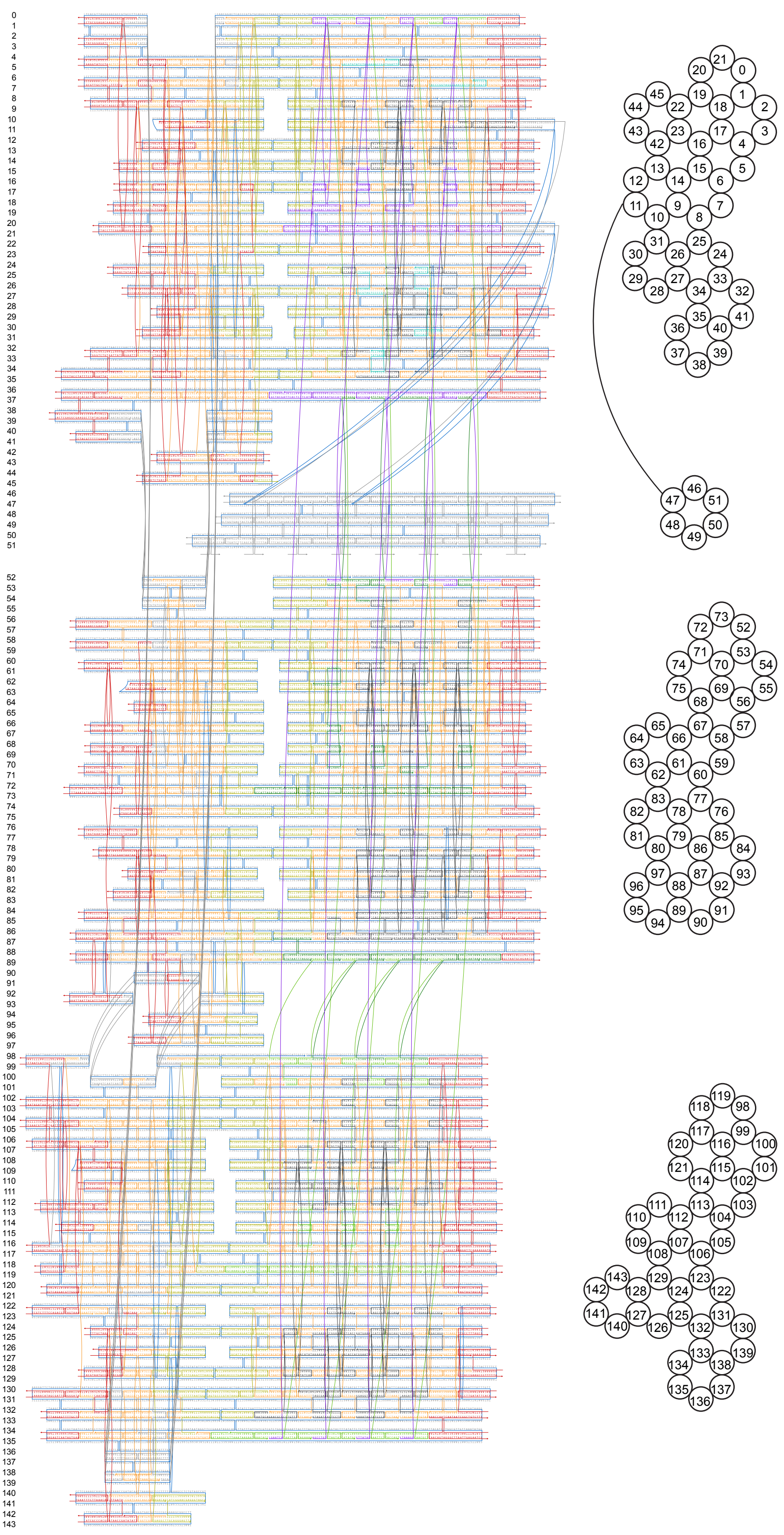

Supplementary Figure 30 | CaDNAno (*1*) design diagram (left) and bottom view cross sections (right) of v6 stator units.

a

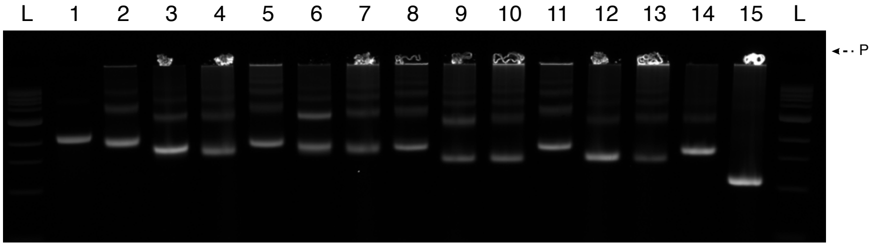

b

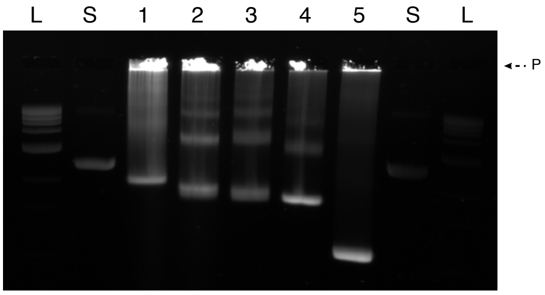

c

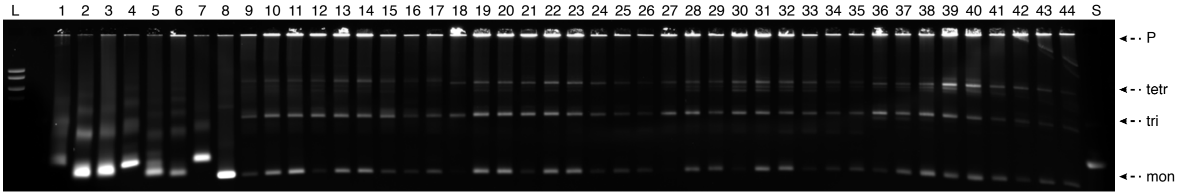

Supplementary Figure 31 | Folding and polymerization of the structure modified to have a stiffer or a more flexible stator. (a) Folding of the different monomers. Laser-scanned photograph of a 2% agarose gel on which the following samples were electrophoresed: L: ladder; S: p8064 scaffold; lane 1: stator unit 1, v2; lane 2: stator unit 2, v2; lane 3: stator unit 3, v2; lane 4: stator unit 1, v4; lane 5: stator unit 2, v4; lane 6: stator unit 3, v4; lane 7: stator unit 1, v3; lane 8: stator unit 2, v3; lane 9: stator unit 3, v3; lane 10: stator unit 1, v6; lane 11: stator unit 2, v6; lane 12: stator unit 3, v6; lane 13: camshaft; lane 14: lever arm. **(b)** Folding of v5. Laser-scanned photograph of a 2% agarose gel on which the following samples were electrophoresed: L: ladder; S: p8064 scaffold; lane 1: stator unit 1; lane 2: stator unit 2; lane 3: stator unit 3; lane 4: camshaft; lane 5: lever arm. **(c)** Polymerization screen of the modified stator variants. Laser-scanned photograph of a 2% agarose gel on which the following samples were electrophoresed: L: ladder; lane 1: stator unit 1, v2; lane 2: stator unit 2, v2; lane 3: stator unit 3, v2; lane 4: stator unit 1, v6; lane 5: stator unit 2, v6; lane 6: stator unit 3, v6; lane 7: camshaft; lane 8: lever arm; lane 9: v2, 30 mM $MgCl_2$, 30°C; lane 10: v2, 40 mM $MgCl_2$, 30°C; lane 11: v2, 50 mM $MgCl_2$, 30°C; lane 12: v2, 30 mM $MgCl_2$, 40°C; lane 13: v2, 40 mM $MgCl_2$, 40°C; lane 14: v2, 50 mM $MgCl_2$, 40°C; lane 15: v2, 30 mM $MgCl_2$, 50°C; lane 16: v2, 40 mM $MgCl_2$, 50°C; lane 17: v2, 50 mM $MgCl_2$, 50°C; lane 18: v4, 30 mM $MgCl_2$, 30°C; lane 19: v4, 40 mM $MgCl_2$, 30°C; lane 20: v4, 50 mM $MgCl_2$, 30°C; lane 21: v4, 30 mM $MgCl_2$, 40°C; lane 22: v4, 40 mM $MgCl_2$, 40°C; lane 23: v4, 50 mM $MgCl_2$, 40°C; lane 24: v4, 30 mM $MgCl_2$, 50°C; lane 25: v4, 40 mM $MgCl_2$, 50°C; lane 26: v4, 50 mM $MgCl_2$, 50°C; lane 27: v3, 30 mM $MgCl_2$, 30°C; lane 28: v3, 40 mM $MgCl_2$, 30°C; lane 29: v3, 50 mM $MgCl_2$, 30°C; lane 30: v3, 30 mM $MgCl_2$, 40°C; lane 31: v3, 40 mM $MgCl_2$, 40°C; lane 32: v3, 50 mM $MgCl_2$, 40°C; lane 33: v3, 30 mM $MgCl_2$, 50°C; lane 34: v3, 40 mM $MgCl_2$, 50°C; lane 35: v3, 50 mM $MgCl_2$, 50°C; lane 36: v6, 30 mM $MgCl_2$, 30°C; lane 37: v6, 40 mM $MgCl_2$, 30°C; lane 38: v6, 50 mM $MgCl_2$, 30°C; lane 39: v6, 30 mM $MgCl_2$, 40°C; lane 40: v6, 40 mM $MgCl_2$, 40°C; lane 41: v6, 50 mM $MgCl_2$, 40°C; lane 42: v6, 30 mM $MgCl_2$, 50°C; lane 43: v6, 40 mM $MgCl_2$, 50°C; lane 44: v6, 50 mM $MgCl_2$, 50°C. P: pockets; pent: pentamers; dim: dimers; mon: monomers.

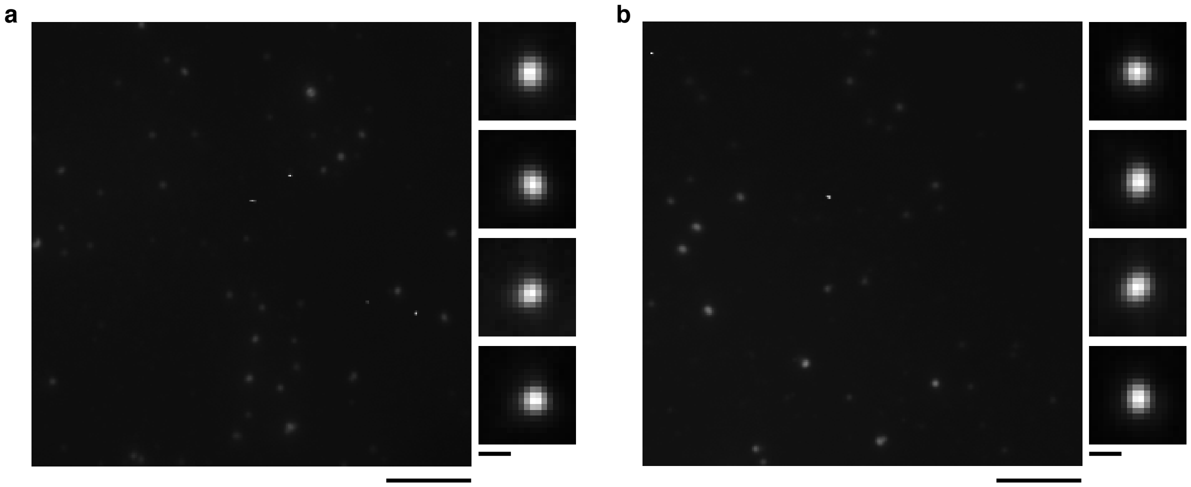

Supplementary Figure 32 | Suppression of the rotation in v4 and v6. **(a)** Typical field of view (left, scale bar: 5 μm) and single-particle standard deviation images (right, scale bar: 600 nm) of v4 in the cy3 channel. **(b)** Typical field of view (left, scale bar: 5 μm) and single-particle standard deviation images (right, scale bar: 600 nm) of v6 in the cy3 channel.

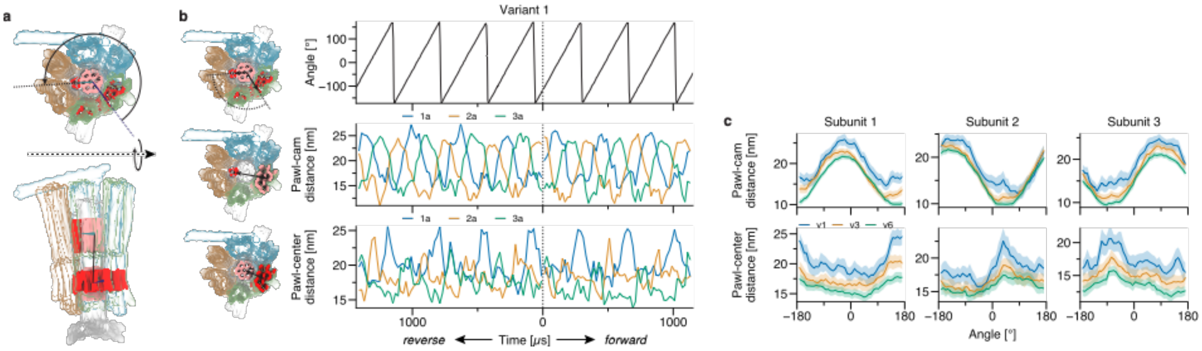

Supplementary Figure 33 | Simulation of driven rotation. (a) Schematic of the dihedral angle used to drive the rotation. To drive the rotation, the rest angle of a harmonic potential applied to this dihedral angle was increased or decreased with a constant rate. **(b)** Forced rotation of variant 1. The time-varying potential acting on the dihedral angle described in panel A caused the rotor to spin (top). The cam cyclically approached each pawl (middle), causing it to deform away from the center of the rotor (bottom). Schematics of each metric are shown to the left of the plots. **(c)** Comparison of forced rotation of variants 1 (blue), 3 (orange) and 6 (green).

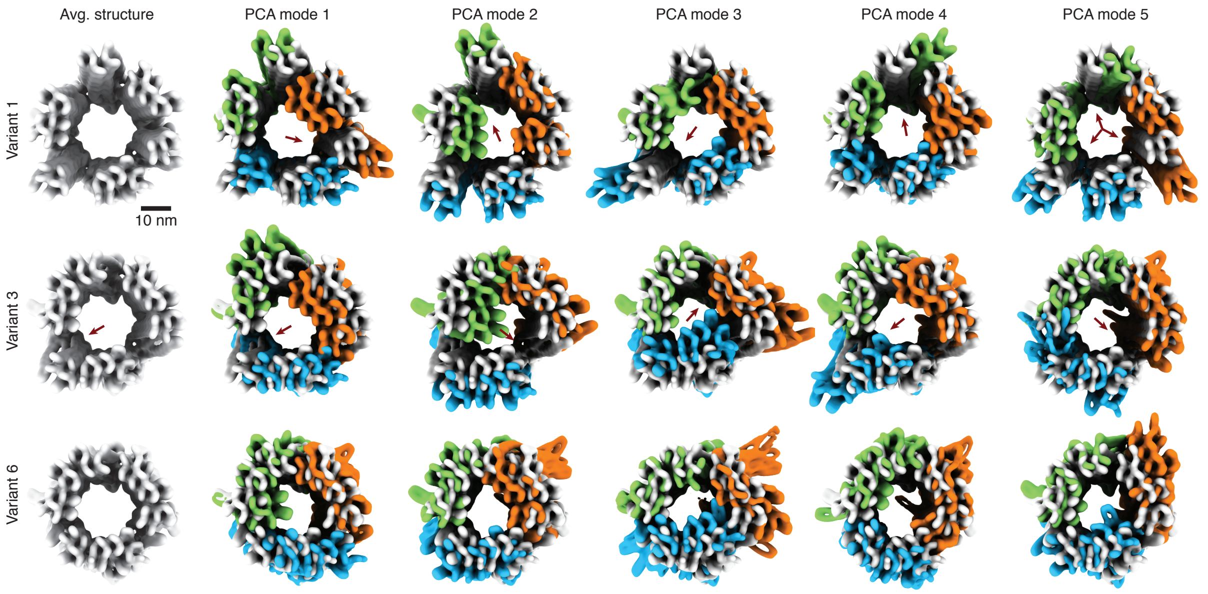

Supplementary Figure 34 | Principal components analysis of pawl configurations. PCA was performed using the scikit-cuda PCA algorithm to extract the top 100 eigenvectors of the cartesian coordinate covariance matrix of each variant 1, 3, and 6 during forced rotation and after aligning the base of the stator. The camshaft was included in the simulations, but excluded from the PCA analysis. The top five modes, accounting for ~50% of the total variance, are visualized here as a deviation (subunits colored blue, orange and green) from the mean structure (white) depicted alone in the first column. The amplitude of the PCA mode deviations was enhanced by the same factor for all modes to make the motion more discernable. Red arrows highlight sites that open up in the PCA mode and are likely to accommodate the rotor cam.

Supplementary Table 1: Cryo-EM maps and EMD codes

Construct name	EMD code	Hyperlinks
Stator unit 1	EMD-13565	https://www.ebi.ac.uk/emdb/EMD-13565
Stator unit 2	EMD-13566	https://www.ebi.ac.uk/emdb/EMD-13566
Stator unit 3	EMD-13567	https://www.ebi.ac.uk/emdb/EMD-13567
Camshaft	EMD-13568	https://www.ebi.ac.uk/emdb/EMD-13568
Empty stator	EMD-13569	https://www.ebi.ac.uk/emdb/EMD-13569
Rotary complex with camshaft bound to stator unit 1	EMD-13570	https://www.ebi.ac.uk/emdb/EMD-13570
Rotary complex with camshaft bound to stator unit 2	EMD-13571	https://www.ebi.ac.uk/emdb/EMD-13571
Rotary complex with camshaft bound to stator unit 3	EMD-13572	https://www.ebi.ac.uk/emdb/EMD-13572
Rotary complex with released camshaft	EMD-13573	https://www.ebi.ac.uk/emdb/EMD-13573

References

1. S. M. Douglas *et al.*, Rapid prototyping of 3D DNA-origami shapes with caDNAno. *Nucleic Acids Res* **37**, 5001-5006 (2009).
