# Supplementary material for: A nanoscale reciprocating rotary mechanism with coordinated mobility control
Source: Nat Commun. 2021 Dec 8;12:7138. doi: 10.1038/s41467-021-27230-7 (PMC8654862; doi:10.1038/s41467-021-27230-7)
Supplement: Supplementary file 2 — Description of Additional Supplementary Files [file 41467_2021_27230_MOESM2_ESM.pdf]

Description of Additional Supplementary Files:

Supplementary Movie 1: Schematic representation of the nanostructure mechanism.

Supplementary Movie 2: Morphed trajectory between the empty stator and the complex with the camshaft bound to the stator unit 1.

Supplementary Movie 3: Coarse grained mrDNA simulations of all six rotor variants with a ~5 bp/bead resolution model. Starting from an idealized geometry taken from the CaDNAno (1) design, each simulation lasted 20 µs.

Supplementary Movie 4: Coarse grained mrDNA simulations of driven rotation of variants 1, 3 and 6 with a ~5 bp/bead resolution model. Starting from an equilibrated configuration, the rotation angle was increased or decreased at a rate of 1 degree per 20 µs. Simulations lasted at least 20 ms, or approximately 3 rotations in either direction.

Supplementary Movie 5: Average configuration of the rotor variants 1, 3 and 6 at a given rotation angle. The coordinates of the rotor were first aligned to minimize the root mean square deviation of the bearing region below the pawls, and were subsequently sorted according to the instantaneous value of the rotation angle into 10° bins. The ensemble of configurations within each bin was averaged and is depicted in the animation. The average includes configuration sampled from both forward and reverse driven rotation simulations.

Supplementary Movie 6: Dominant principal component analysis modes of the stator for variants 1, 3 and 6. The white surface depicts the average structure. PCA modes determined as described for Supplementary Figure 34.

Description of Supplementary Data file

Supplementary Data 1: Sequences for stator unit 1 (SU1) used for cryo analysis. Scaffold sequence named *scaffold.*

Staple names	SU1 for cryo – binding to CS	SU1 for cryo – not binding to CS
SU1_core	yes	/
SU1_noBind_core	/	yes
SU1_end	yes	yes
SU1_joint	yes	yes
SU1_interface	yes	yes
SU1toSU2_sticky	yes	yes
SU1toSU3_sticky	yes	yes
SU1_incumbent	yes	/

Supplementary Data 2: Sequences for all stator units 1 used for TIRFM analysis (SU1T). Scaffold sequence named *scaffold.* Biotinylated oligo named biotin_01.

Staple names	V1	V2	V3	V4	V5	V6	Bound to 6hb
SU1T_corev1	yes	yes	/	/	/	/	yes
SU1T_corev3	/	/	yes	/	/	/	/
SU1T_corev4	/	/	/	yes	yes	/	/
SU1T_corev6	/	/	/	/	/	yes	/
SU1T_end	yes	yes	yes	yes	yes	yes	yes
SU1T_overhang	yes	yes	yes	yes	yes	yes	/
SU1T_bound_overhang	/	/	/	/	/	/	yes
SU1T_interface	yes	/	yes	yes	yes	yes	yes
SU1T_interfacev2	/	yes	/	/	/	/	/
SU1T_incumbent	yes	yes	yes	yes	yes	yes	yes
SU1T_joint	yes	yes	yes	/	/	/	yes
SU1T_jointv4	/	/	/	yes	/	yes	/
SU1T_jointv5	/	/	/	/	yes	/	/
SU1T_biotinAnhcor	yes	yes	yes	yes	yes	yes	/
SU1T_bound_biotinAnhcor	/	/	/	/	/	/	yes
SU1T_body	yes	yes	yes	yes	yes	yes	/
SU1T_bound_body	/	/	/	/	/	/	yes
SU1T_to_SU2_sticky	yes	yes	yes	yes	yes	yes	yes
SU1T_to_SU3_sticky	yes	yes	yes	yes	yes	yes	yes
SU1T_fluo	yes	yes	yes	yes	yes	yes	/
SU1T_to_6hb_sticky	/	/	/	/	/	/	yes

Supplementary Data 3: Sequences for all stator units 2 (SU2). Scaffold sequence named *scaffold.*

Staple names	SU2 cryo – not bound to CS	SU2 cryo – bound to CS	V1	V2	V3	V4	V5	V6
SU2_core	yes	/	yes	yes	/	/	/	/
SU2_CSbound_core	/	yes	/	/	/	/	/	/
SU2_corev3	/	/	/	/	yes	/	/	/
SU2_corev4	/	/	/	/	/	yes	yes	/
SU2_corev6	/	/	/	/	/	/	/	yes
SU2_end	yes	yes	yes	yes	yes	yes	yes	yes
SU2_interface	yes	yes	yes	/	yes	yes	yes	yes
SU2_interfacev2	/	/	/	yes	/	/	/	/
SU2_joint	yes	yes	yes	yes	yes	/	/	/
SU2_jointv4	/	/	/	/	/	yes	/	yes
SU2_jointv5	/	/	/	/	/	/	yes	/
SU2_to_SU3_sticky	yes	yes	yes	yes	yes	yes	yes	yes
SU2_to_SU1_sticky	yes	yes	/	/	/	/	/	/
SU2_to_SU1T_sticky	/	/	yes	yes	yes	yes	yes	yes
SU2_incumbent	/	yes	/	/	/	/	/	/

Supplementary Data 4: Sequences for all stator units 3 (SU3). Scaffold sequence named *scaffold.*

Staple names	SU3 cryo – not bound to CS	SU3 cryo – bound to CS	V1	V2	V3	V4	V5	V6
SU3_core	yes	/	yes	yes	/	/	/	/
SU3_CSbound_core	/	yes	/	/	/	/	/	/
SU3_corev3	/	/	/	/	yes	/	/	/
SU3_corev4	/	/	/	/	/	yes	yes	/
SU3_corev6	/	/	/	/	/	/	/	yes
SU3_end	yes	yes	yes	yes	yes	yes	yes	yes
SU3_interface	yes	yes	yes	/	yes	yes	yes	yes
SU3_interfacev2	/	/	/	yes	/	/	/	/
SU3_joint	yes	yes	yes	yes	yes	/	/	/
SU3_jointv4	/	/	/	/	/	yes	/	yes
SU3_jointv5	/	/	/	/	/	/	yes	/
SU3_to_SU2_sticky	yes	yes	yes	yes	yes	yes	yes	yes
SU3_to_SU1_sticky	yes	yes	/	/	/	/	/	/
SU3_to_SU1T_sticky	/	/	yes	yes	yes	yes	yes	yes
SU3_incumbent	/	yes	/	/	/	/	/	/

Supplementary Data 5: Sequences for the camshaft (CS). Scaffold sequence named *scaffold*. Invader strands to release camshaft from stator named *invader*.

Staple names	CS	CS – binding to LA
CS_core	yes	yes
CS_end	yes	/
CS_LA_end	/	yes

Supplementary Data 6: Sequences for lever arm, named LA_core, LA_ends, LA_fluo. Scaffold sequence named *scaffold.*

Supplementary Data 7: Sequences for 6hb pointer, named 6hb_core, 6hb_fluo. Scaffold sequence named *scaffold.*

Supplementary Data 8: Uncropped gel scans.
