# Supplementary material for: A nanoscale reciprocating rotary mechanism with coordinated mobility control
Source: Nat Commun. 2021 Dec 8;12:7138. doi: 10.1038/s41467-021-27230-7 (PMC8654862; doi:10.1038/s41467-021-27230-7)
Supplement: Supplementary file 17 — Supplementary Data 8 [file 41467_2021_27230_MOESM17_ESM.pdf]

A nanoscale reciprocating rotary mechanism with coordinated mobility control

Eva Bertosin[1], Christopher M. Maffeo[2,3], Thomas Drexler[1], Maximilian N. Honemann[1], Aleksei Aksimentiev[2,3], Hendrik Dietz[1]

[1]Lehrstuhl für Biomolekulare Nanotechnologie, Physik Department, Technische Universität München, Garching near Munich, Germany

[2]Department of Physics, University of Illinois at Urbana-Champaign, Urbana, IL, 61801, USA

[3]Center for Macromolecular Modeling and Bioinformatics, Beckman Institute for Advanced Science and Technology, University of Illinois at Urbana-Champaign, Urbana, IL, 61801, USA

Correspondence to dietz@tum.de

Supplementary Data 1

Content

Uncropped gel scans

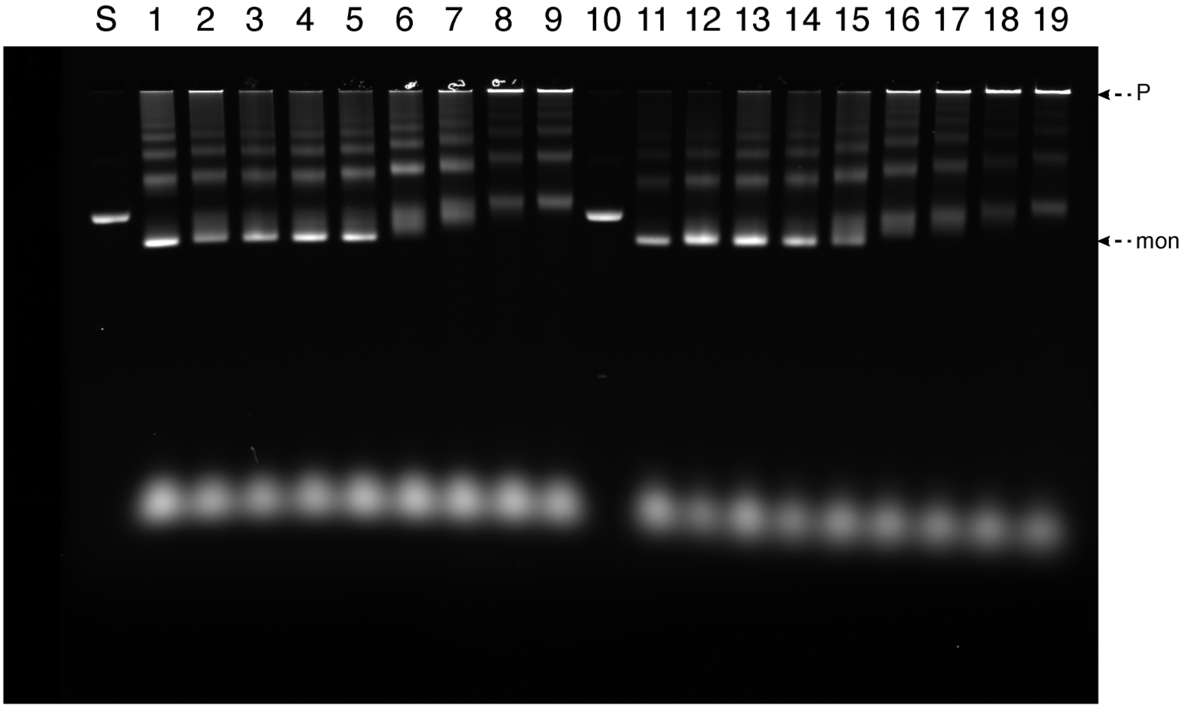

Supplementary Data Figure 1 Uncropped gel corresponding to Supplementary Figure 4a.

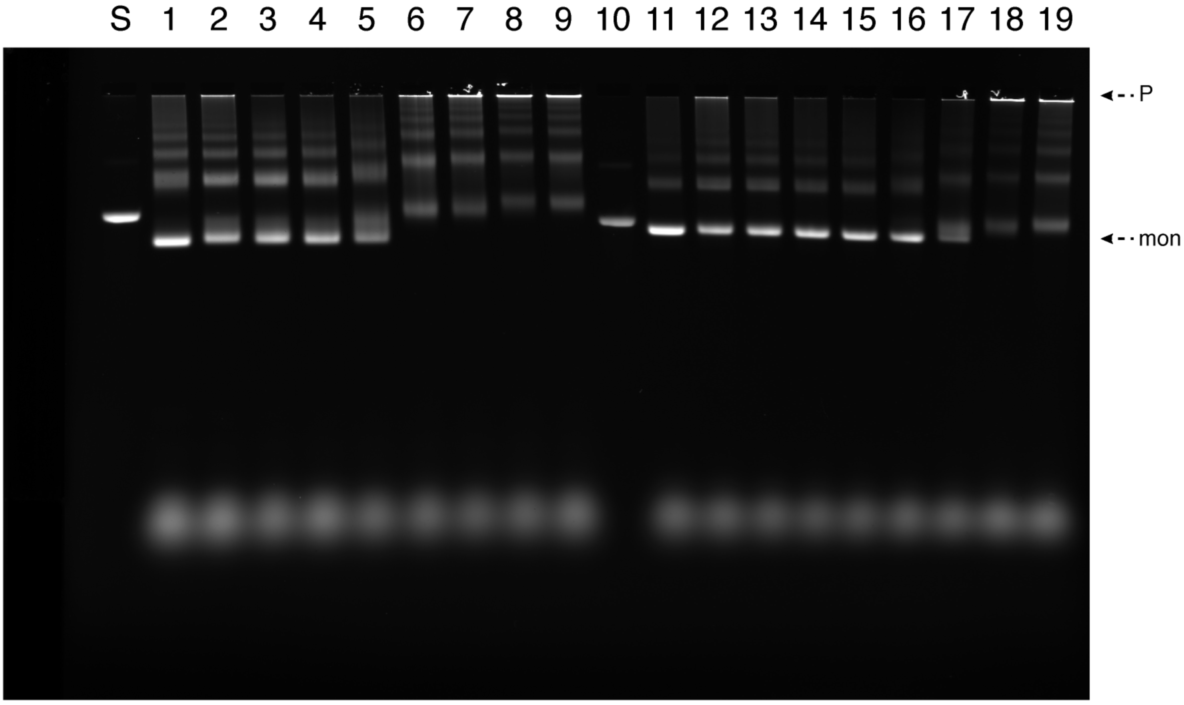

Supplementary Data Figure 2 Uncropped gel corresponding to Supplementary Figure 4b.

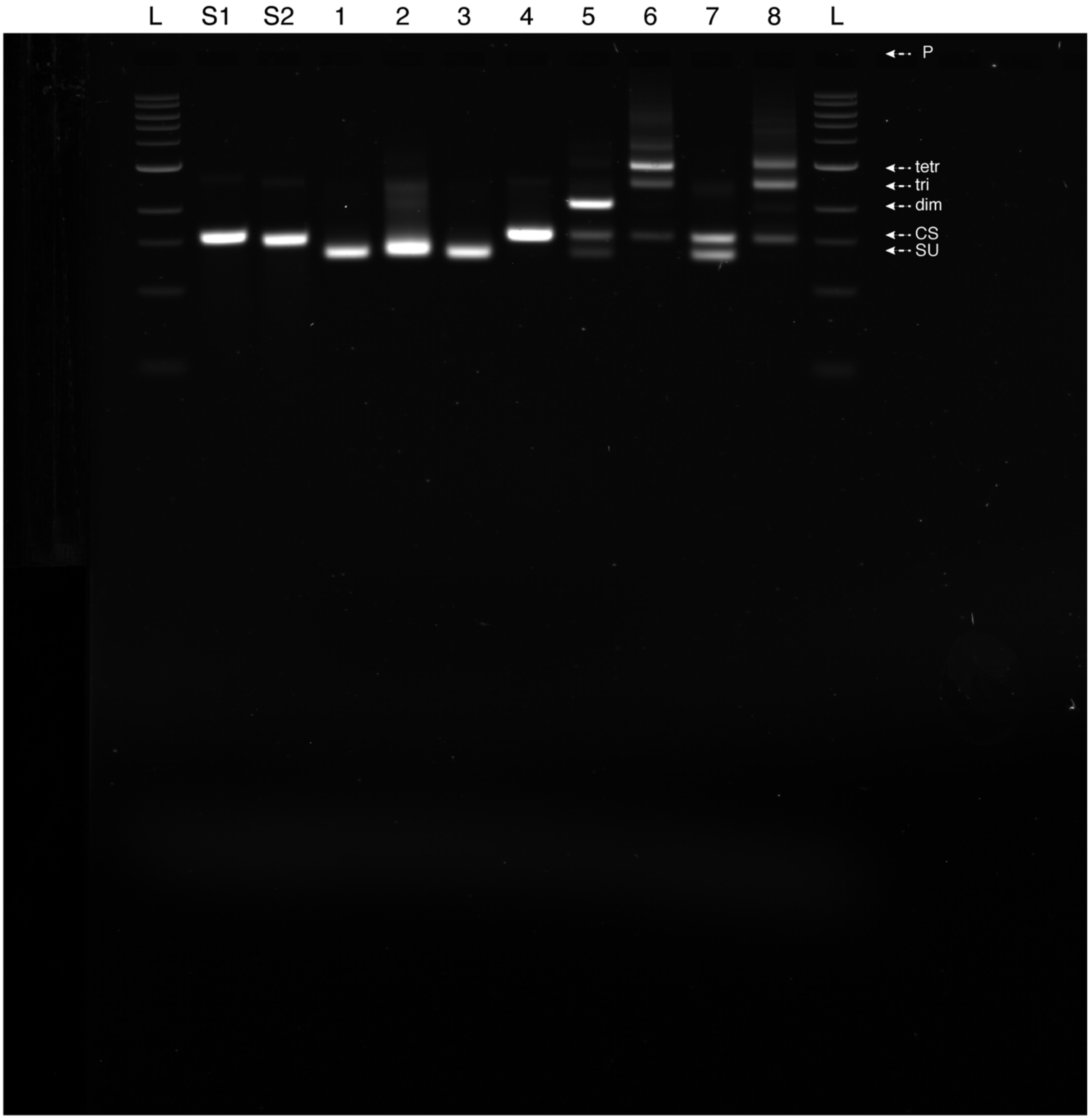

Supplementary Data Figure 3 Uncropped gel corresponding to Supplementary Figure 5a.

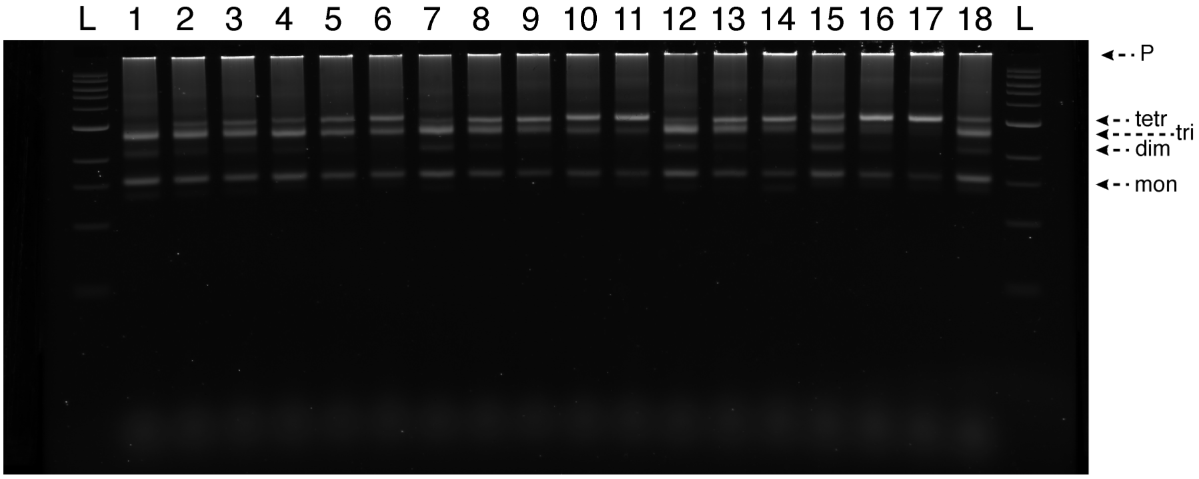

Supplementary Data Figure 4 Uncropped gel corresponding to Supplementary Figure 5b.

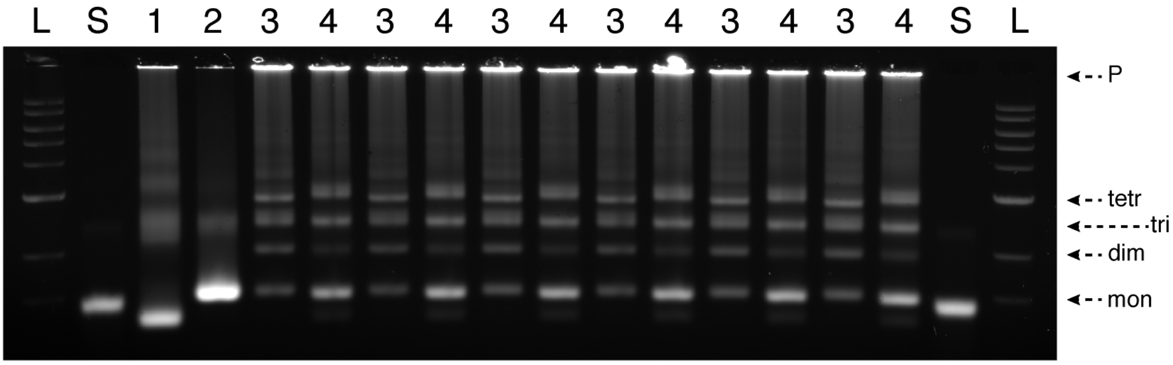

Supplementary Data Figure 5 Uncropped gel corresponding to Supplementary Figure 5c.

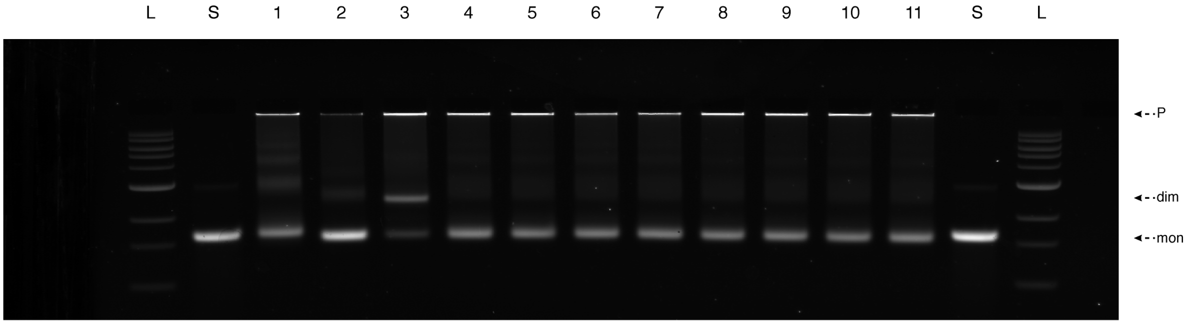

Supplementary Data Figure 6 Uncropped gel corresponding to Supplementary Figure 17a.

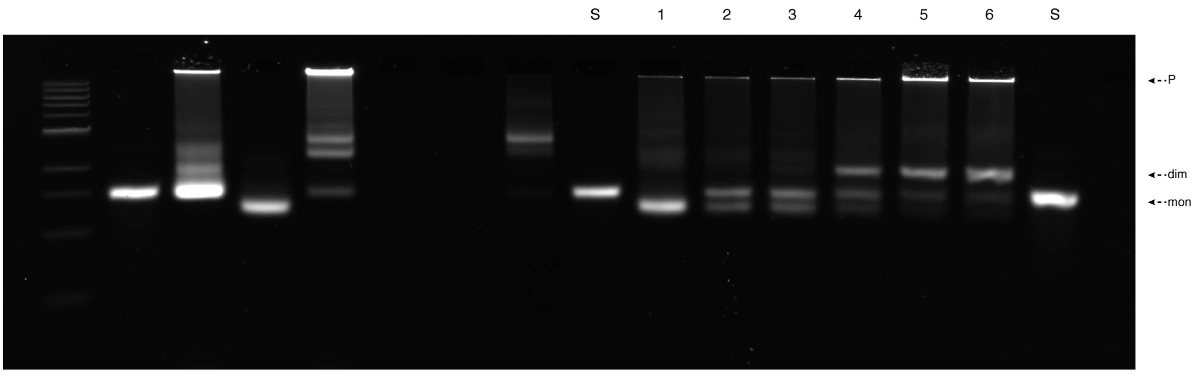

Supplementary Data Figure 7 Uncropped gel corresponding to Supplementary Figure 17b.

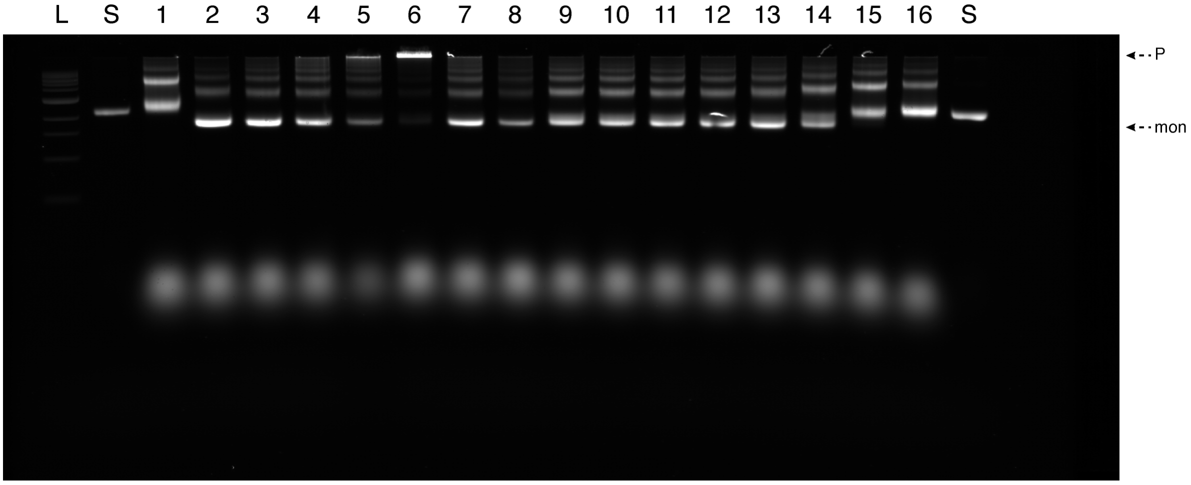

Supplementary Data Figure 8 Uncropped gel corresponding to Supplementary Figure 20.

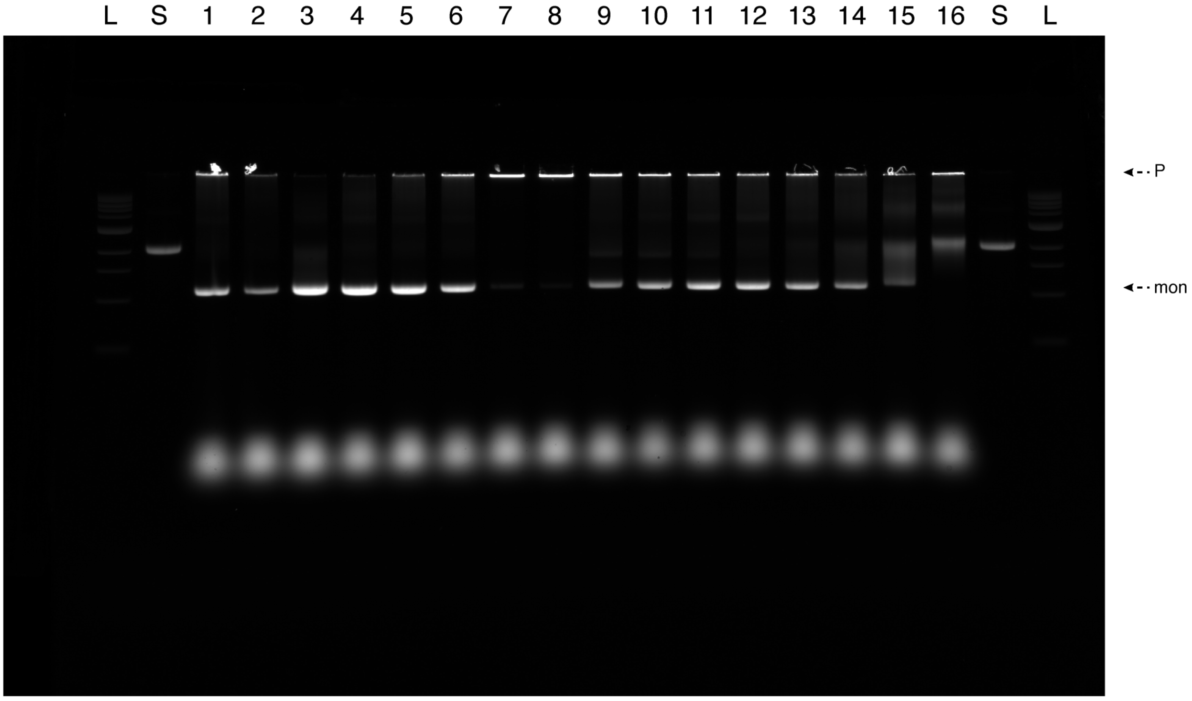

Supplementary Data Figure 9 Uncropped gel corresponding to Supplementary Figure 22a.

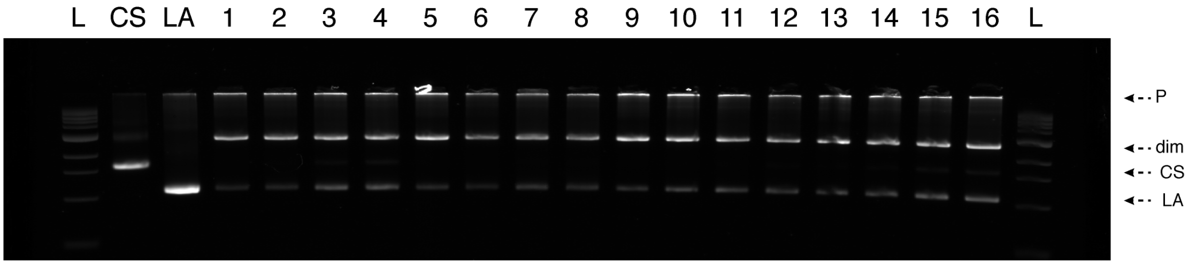

Supplementary Data Figure 10 Uncropped gel corresponding to Supplementary Figure 22b.

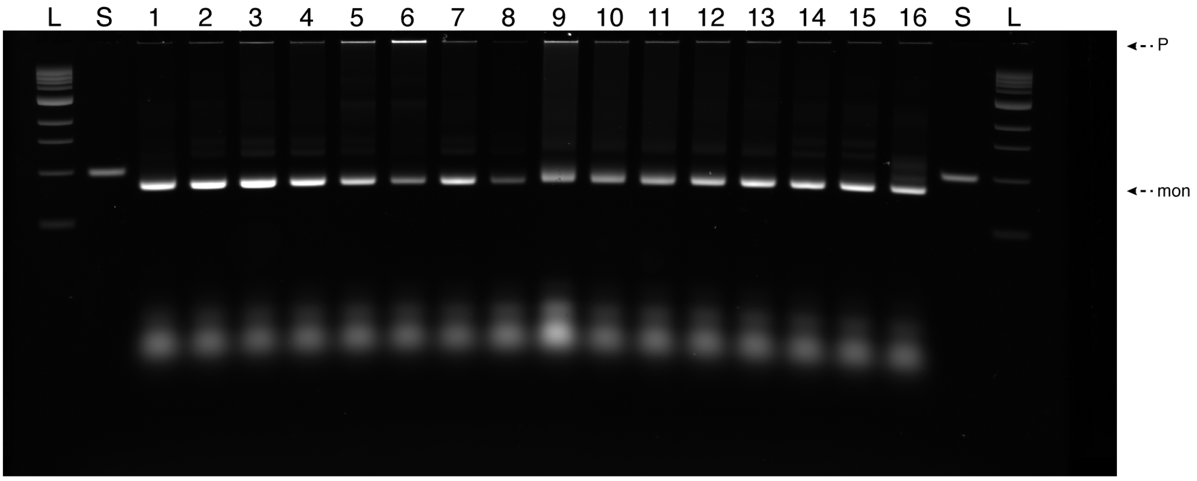

Supplementary Data Figure 11 Uncropped gel corresponding to Supplementary Figure 24a.

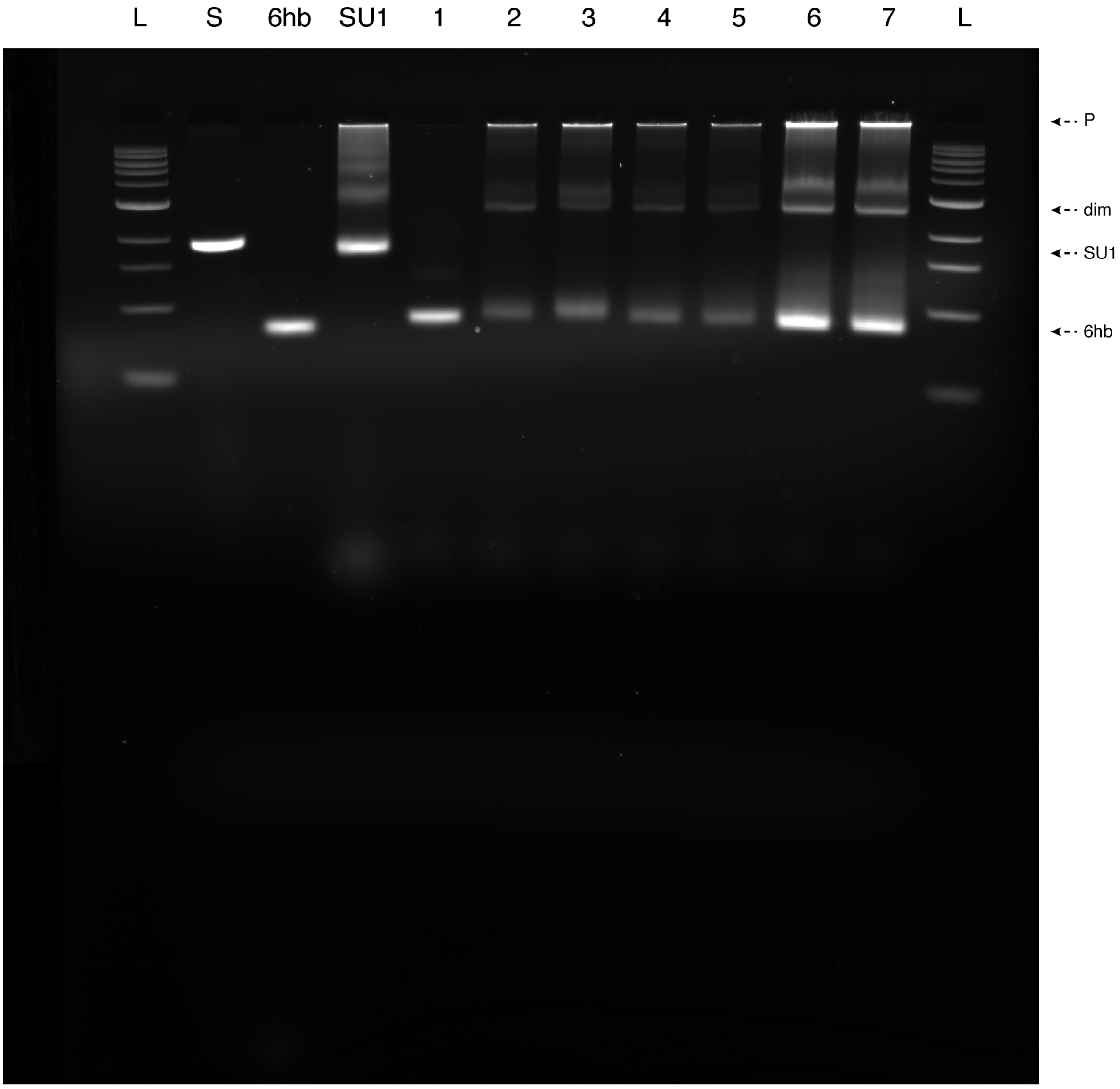

Supplementary Data Figure 12 Uncropped gel corresponding to Supplementary Figure 24b.

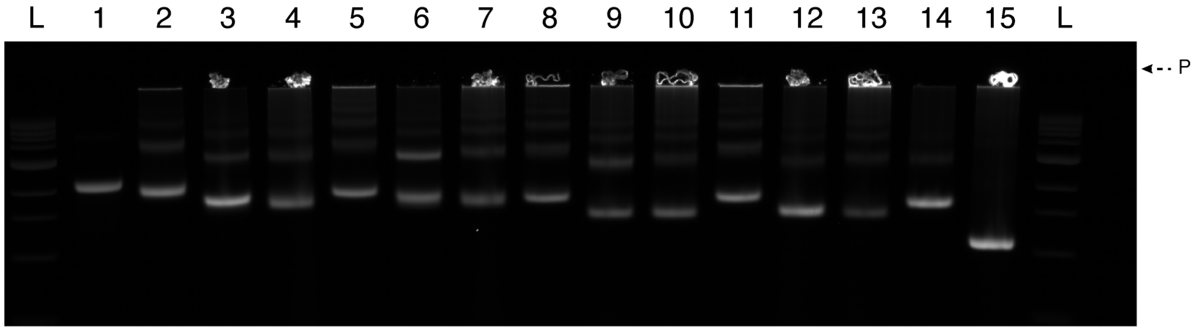

Supplementary Data Figure 13 Uncropped gel corresponding to Supplementary Figure 31a.

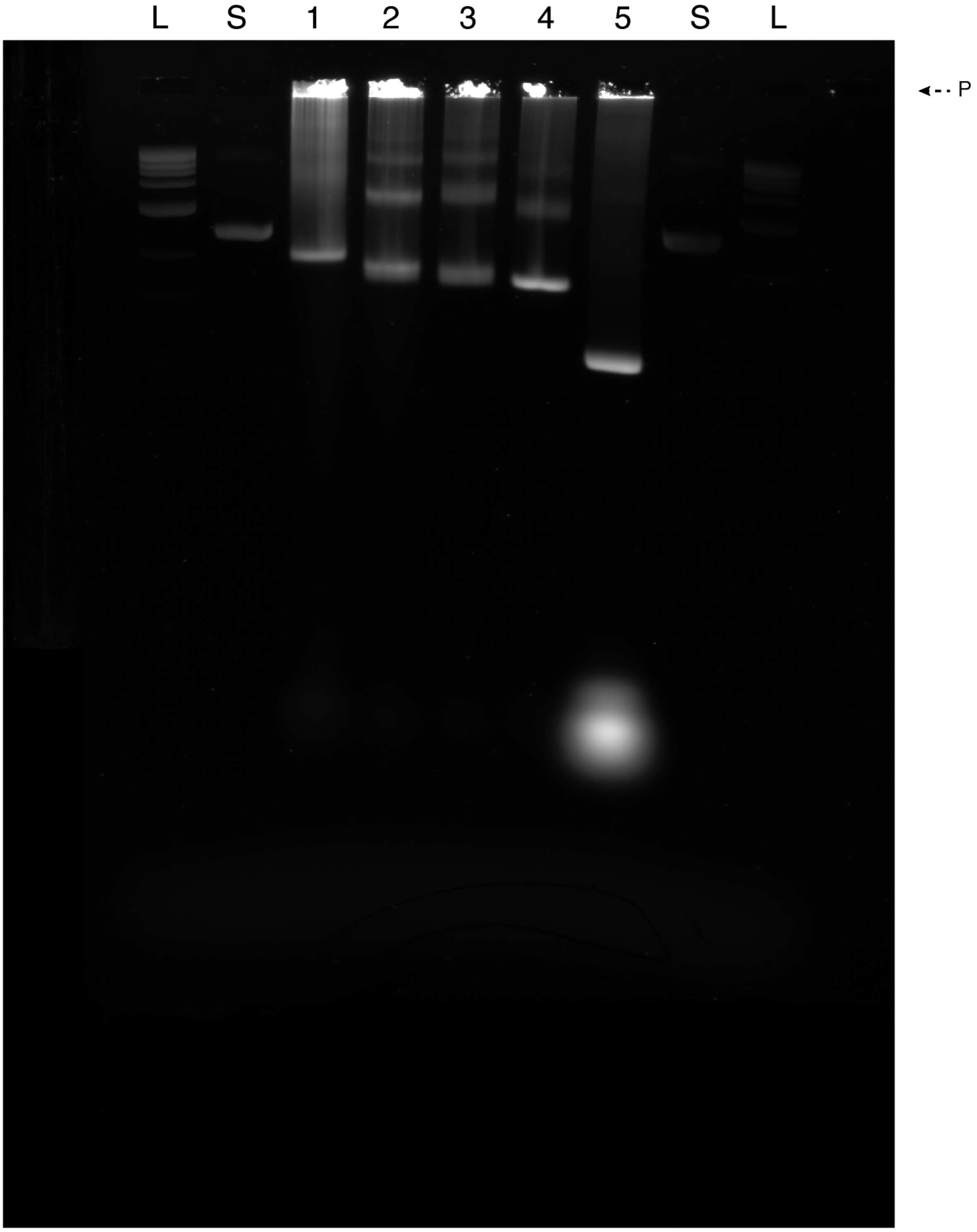

Supplementary Data Figure 14 Uncropped gel corresponding to Supplementary Figure 31b.

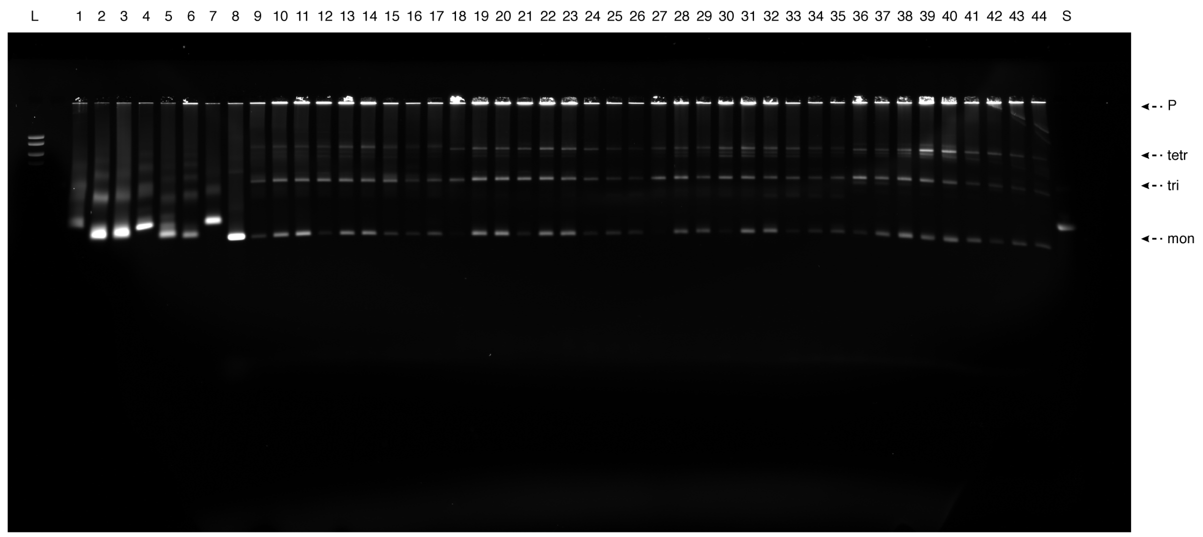

Supplementary Data Figure 15 Uncropped gel corresponding to Supplementary Figure 31c.
